# Conservation of streptococcal CRISPRs on human skin and saliva

**DOI:** 10.1186/1471-2180-14-146

**Published:** 2014-06-06

**Authors:** Refugio Robles-Sikisaka, Mayuri Naidu, Melissa Ly, Julia Salzman, Shira R Abeles, Tobias K Boehm, David T Pride

**Affiliations:** 1 Department of Pathology, University of California, San Diego, 9500 Gilman Drive, MC 0612, La Jolla, CA 92093-0612, USA; 2 Department of Biochemistry, Stanford University School of Medicine, 279 Campus Drive, Stanford, CA 94305-5329, USA; 3 Department of Medicine, University of California, San Diego, 9500 Gilman Drive, MC 0612, La Jolla, CA 92093-0612, USA; 4 College of Dental Medicine, Western University of Health Sciences, 309 E Second Street, Pomona, CA 91766, USA

**Keywords:** CRISPR, Skin microbiome, Saliva microbiome, Virome, Virus

## Abstract

**Background:**

Clustered Regularly Interspaced Short Palindromic Repeats (CRISPRs) are utilized by bacteria to resist encounters with their viruses. Human body surfaces have numerous bacteria that harbor CRISPRs, and their content can provide clues as to the types and features of viruses they may have encountered.

**Results:**

We investigated the conservation of CRISPR content from streptococci on skin and saliva of human subjects over 8-weeks to determine whether similarities existed in the CRISPR spacer profiles and whether CRISPR spacers were a stable component of each biogeographic site. Most of the CRISPR sequences identified were unique, but a small proportion of spacers from the skin and saliva of each subject matched spacers derived from previously sequenced loci of *S. thermophilus* and other streptococci. There were significant proportions of CRISPR spacers conserved over the entire 8-week study period for all subjects, and salivary CRISPR spacers sampled in the mornings showed significantly higher levels of conservation than any other time of day. We also found substantial similarities in the spacer repertoires of the skin and saliva of each subject. Many skin-derived spacers matched salivary viruses, supporting that bacteria of the skin may encounter viruses with similar sequences to those found in the mouth. Despite the similarities between skin and salivary spacer repertoires, the variation present was distinct based on each subject and body site.

**Conclusions:**

The conservation of CRISPR spacers in the saliva and the skin of human subjects over the time period studied suggests a relative conservation of the bacteria harboring them.

## Background

Viruses form a substantial portion of the human microbiome, and many have previously been identified as bacteriophage living in association with the numerous cellular microbes that inhabit human body surfaces [1-4]. Relative to their bacterial counterparts, there have been comparatively few studies characterizing human viral communities [3-9]. Many of these studies of human viruses generally have been limited to cross-sectional analyses, where little could be ascertained about the stability or the rate of turnover of viruses in these environments. Moreover, the effects of environment on the composition of human viral communities have not been thoroughly examined. We recently demonstrated that individuals living together are significantly more likely to have similar oral viruses [10].

CRISPRs (Clustered Regularly Interspaced Short Palindromic Repeats) are part of the CRISPR/Cas system in bacteria and archaea and mediate an adaptive immune response against invading viruses. They function by acquiring short sequences from invading viruses into the CRISPR locus, and counteract future infections through nucleic acid interference [11-13]. Because CRISPR loci acquire and accumulate short viral sequences, they have been used to trace viral exposures [14-18]. In addition to having similar oral viruses, household members also have significant similarities in their CRISPR spacer profiles [10], suggesting that oral CRISPR spacers may evolve as a result of each individual’s oral virome composition.

While certain human sample types such as saliva, feces, and respiratory secretions provide sufficient biomass to characterize viral populations [1,3,4,19], the skin generally presents a more challenging body surface on which to characterize viruses. Because of a general lack of starting material, analysis of the skin microbiome mostly has been limited to analysis of those microbes on skin swabs or scrapings [20-22]. To analyze skin viral populations, Foulongne *et al.* recently used high-throughput sequencing techniques to sequence the skin metagenome, and to analyze those viruses present by targeted analysis of viral reads [23]. In most human sample types, the majority of the viruses present have been identified as bacteriophage [1-3,19], which may reflect the 10 to 1 proportion of bacterial to human cells in these environments. In analysis of the skin virome, however, bacteriophage constituted only a small proportion of the metagenome sequences [23]. By examining the CRISPR spacer profiles of the skin, we may improve our understanding of the sequence features of viruses to which skin bacteria have previously encountered.

Study of the human microbiome has detailed unique populations of microbes inhabiting different body surfaces. While the oral cavity and the skin surfaces differ substantially in their bacterial constituents, they share some bacterial genera including some species from the genus *Streptococcus*[24]. Streptococci generally are present on the skin and in the saliva of most humans [25-28], and represent a substantial proportion of the oral microbiota and a much smaller proportion of the skin microbiota [29-33]. The human oral cavity is known to harbor various types of viridans streptococci, including *S. mutans*, *S. gordonii*, *S. oralis*, *S. mitis*, *S. milleri* (includes *S. anginosus*, *S. constellatus*, and *S. intermedius*), *S. sanguinis*, and *S. parasanguinis*, and also some non-viridans streptococci, including *S. bovis* (includes *S. gallolyticus*, *S. equinus,* and *S. infantarius*, among others). The skin generally harbors different species of streptococci, including *S. pyogenes* and *S. agalactiae*, which belong to Lancefield groups A and B, respectively. The skin also is known to harbor streptococci that belong to Lancefield groups C and G [24]. In this study, we sought to characterize the CRISPR profiles present in a cohort of human subjects on both their skin and in their oral cavities. Our goals were to determine whether there were similar CRISPR profiles among streptococci on human skin and saliva, whether CRISPR content on the skin and saliva was relatively conserved over time, and whether there were CRISPR spacers present on human skin that matched viruses present in saliva.

## Results

### CRISPR spacer sequencing

We sampled 4 human subjects with good overall cutaneous and periodontal health, collecting skin swabs and saliva samples 3 times per day on days #1, #2, #4, #14, #28, and week #8. Skin and saliva samples were collected at the same time in the AM prior to breakfast or oral hygiene (AM), approximately noon each day before lunch (Noon), and in the early evening prior to dinner [34]. Skin samples were collected from the volar surface of the forearm, as prior studies utilizing this surface provide insight into its indigenous microbes [31,33,35]. We analyzed Streptococcus Group I (SGI) and Streptococcus Group II (SGII) CRISPRs, by amplifying them based on their consensus repeat motifs (Additional file 1: Table S1) [14,15]. These CRISPR repeat motifs are present in a variety of different streptococcal species, including *S. pyogenes* and *S. agalactiae* that are primarily found on the skin, and numerous different viridans streptococci such as *S. mutans*, *S. gordonii*, *S. mitis*, and *S. sanguinis* that are found in the oral cavity (Additional file 1: Table S2). The benefits of this approach were that we could analyze CRISPR spacers from numerous streptococcal species simultaneously and were not limited to examining individual CRISPR loci. The main drawbacks of this technique were that it was difficult to ascribe the spacers to any single CRISPR locus or bacterial species, and the consensus repeat motifs could be present in some non-streptococcal species. We amplified CRISPRs from all subjects, sample types, and time points, and sequenced 4,090,937 CRISPR spacers consisting of 2,212,912 SGI and 1,878,025 SGII spacers using semiconductor sequencing [36] (Additional file 1: Table S3). There were 2,169,768 spacers obtained from saliva and 1,921,169 spacers obtained from skin. For all time points combined, we found 1,055,321 spacers for Subject #1, 781,534 spacers for Subject #2, 1,088,339 for Subject #3, and 891,618 spacers for Subject #4.

### Spacer binning and estimated coverage

We binned each of the CRISPR spacers according to trinucleotide content according to our previously described protocols [10]. The majority of the CRISPR spacers identified in each subject and time point were identical to other spacers, with only 0.001% of SGI and 0.002% of SGII spacers identified as having polymorphisms that necessitated grouping according to trinucleotide content. We sequenced an average of 28,333 spacers per time point and sample type in each subject to capture the majority of the CRISPR spacer diversity in these environments. We then performed rarefaction analysis on all subjects by CRISPR and sample type to estimate how thoroughly each had been evaluated. We found that all curves neared asymptote for all subjects, sample types, and time points, with the exception of Subject#1 in the evening of week 8 for SGII CRISPR spacers (Additional file 2: Figure S1).

### CRISPR spacer distribution

**Figure 1:**
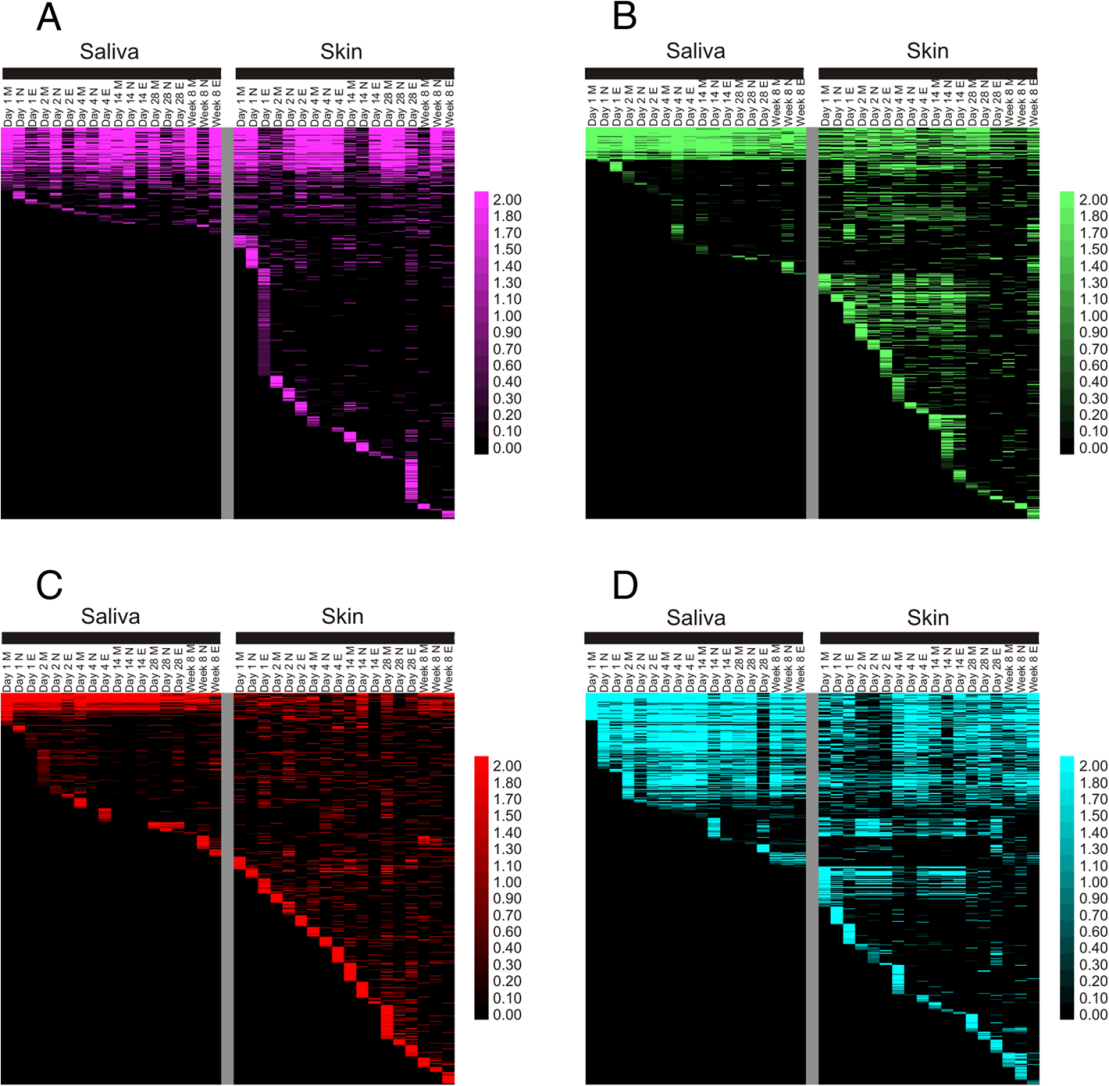
**Heatmaps of SGI CRISPR spacer groups in all subjects.** Each row represents a unique spacer group and the columns represent each individual time point. Each day is listed, where M represents morning, N represents noon, and E represents evening. Saliva-derived SGI CRISPR spacer groups are demonstrated on the left, and skin-derived CRISPR spacer groups are on the right of each panel. The intensity scale bar is located to the right, and represents the percentage of total spacers found at each time point in each subject. Panel **A** – Subject #1, Panel **B** – Subject #2, Panel **C** – Subject #3, and Panel **D** – Subject #4.

**Figure 2:**
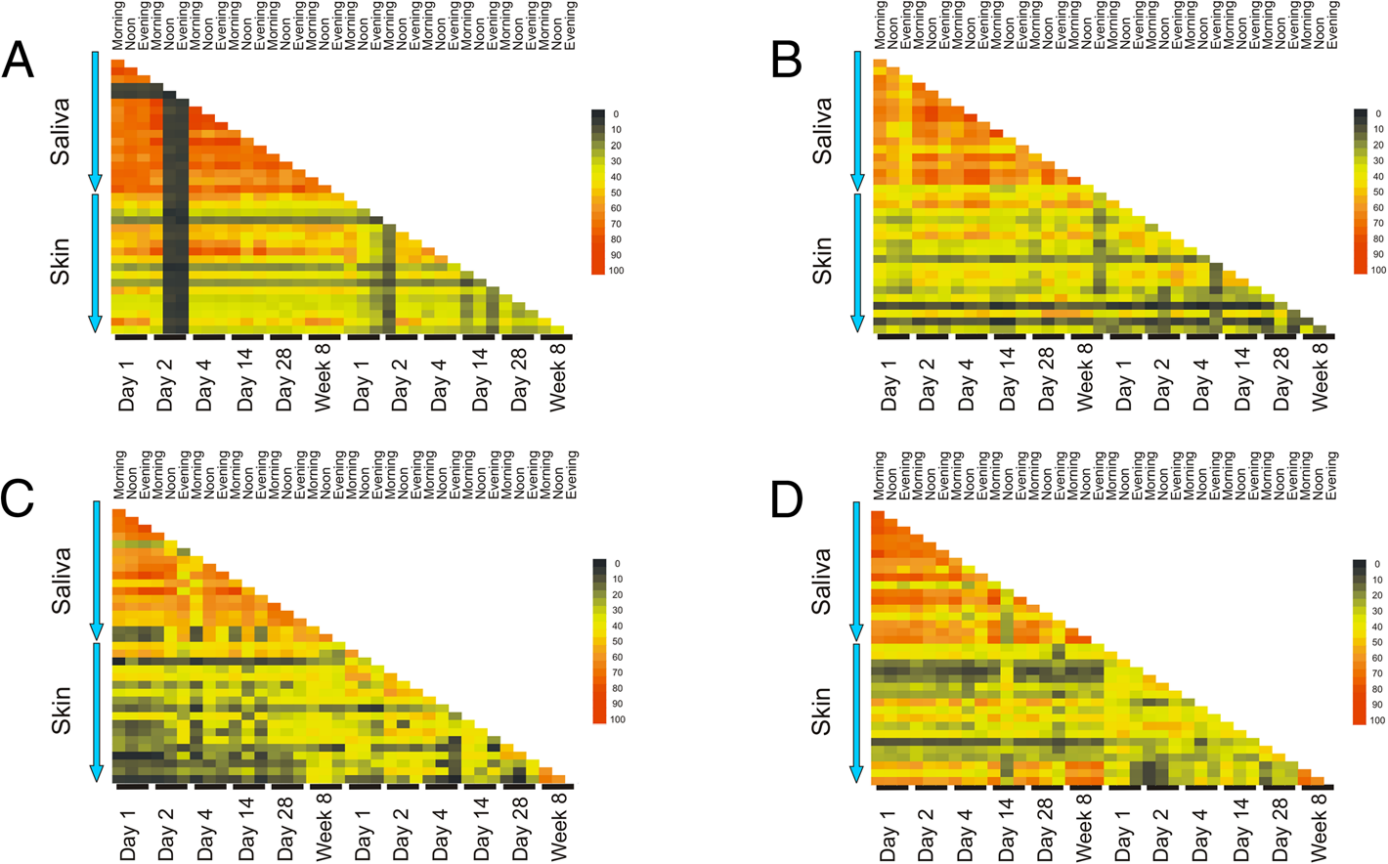
**SGI CRISPR spacer group heat matrices from all subjects.** Each matrix demonstrates the percentage of shared SGI CRISPR spacer groups between all time points within each subject. The top triangular portion of each matrix represents comparisons between saliva-derived CRISPR spacers, the bottom rectangular portion of each matrix represents comparisons between saliva-derived and skin-derived CRISPR spacers, and the bottom triangular portion of each matrix represents comparisons between skin-derived CRISPR spacers. The intensity scale bar is located to the right of each matrix. Panel **A** – Subject #1, Panel **B** – Subject #2, Panel **C** – Subject #3, and Panel **D** – Subject #4.

We compared CRISPR spacers and their relative abundances across all time points in each subject to determine how spacers in each subject were distributed over time. At each time point, many of the spacers found at early time points persisted throughout later time points (Figure 1 and Additional file 2: Figure S2), indicating that many of the SGI and SGII CRISPR spacers were conserved throughout the study period. We quantified the proportions of persistent SGI salivary spacers and found 61% to be persistent in Subject #1, 62% in Subject #2, 36% in Subject #3, and 49% in Subject #4 (Figure 2 and Additional file 1: Table S4). This was similar for SGII salivary spacers (45% persistent in Subject #1, 65% in Subject #2, 51% in Subject #3, and 58% in Subject #4) (Additional file 2: Figure S3 and Additional file 1: Table S4). There was a smaller yet similar group of spacers on the skin of each subject for SGI spacers (38% in Subject #1, 36% in Subject #2, 15% in Subject #3, and 24% in Subject #4) and SGII spacers (39% in Subject #1, 28% in Subject #2, 10% in Subject #3, and 36% in Subject #4) persisting throughout the study. Many of the conserved spacers in saliva matched spacers on the skin of each subject for SGI spacers (44% in Subject #1, 41% in Subject #2, 11% in Subject #3, and 25% in Subject #4) and SGII spacers (42% in Subject #1, 30% in Subject #2, 17% in Subject #3, and 37% in Subject #4).

**Figure 3:**
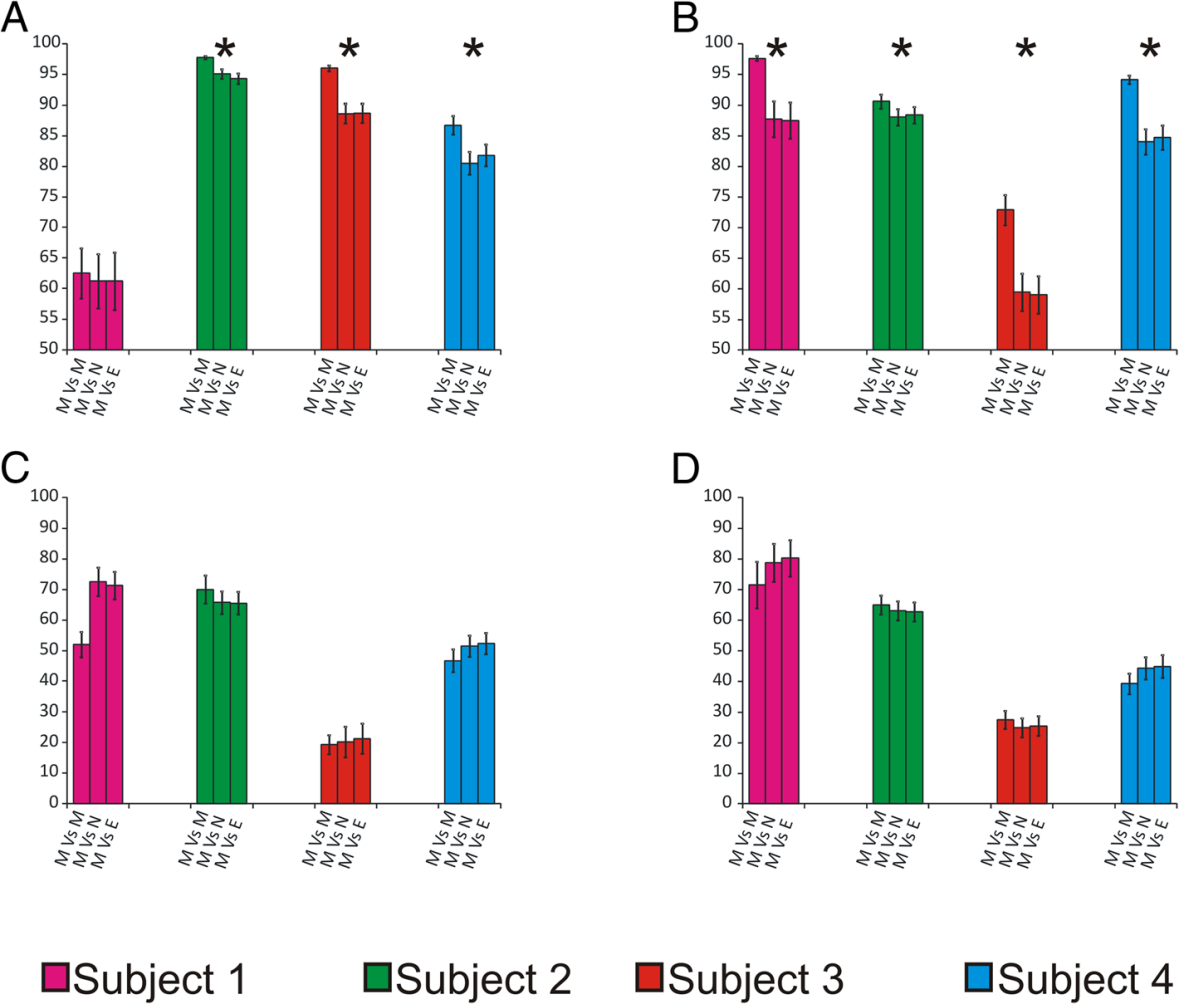
**Conservation of CRISPR spacer content by time of day sampled.** Each panel demonstrates the relative conservation of spacers (±standard deviation) within the morning time points for each subject (M vs. M), comparisons of the morning time points with noon time points (M vs. N), and comparisons of the morning time points with the evening time points (M vs E) for subject #1 (magenta), subject #2 [22], subject #3 (red), and subject #4 (cyan). Panels **A** and **B** represent salivary SGII and SGI CRISPR spacers, respectively. Panels **C** and **D** represent skin-derived SGII and SGI CRISPR spacers, respectively. The '*' represents subjects in which the relative conservation of spacers for the morning time points is significantly (p ≤ 0.05) greater than for comparisons of morning and noon/evening time points.

We measured the relative conservation of SGII and SGI spacers by time of day sampled to determine whether there were biases in CRISPR spacer profiles on the skin and in the saliva based on sampling times. We found that in the saliva, there was significantly greater conservation (p < 0.05) of CRISPR spacer profiles in the AM for both SGII (Figure 3, Panel A) and SGI spacers (Panel B). Similar conservation of CRISPR spacer profiles were not found for Noon and PM time points for either SGII or SGI spacers in saliva (Additional file 2: Figures S4 and S5). Because the saliva samples were collected prior to meals and oral hygiene practices in the AM, while the Noon sample was collected after breakfast and oral hygiene and the PM time point was collected after lunch, we believe that the substantial time between disturbances for the AM time points resulted in greater stability of the oral bacteria harboring these CRISPR loci. Similar results were not found on the skin for any time points (Figure 3, Panels B and C, and Additional file 2: Figures S4 and S5). We did not control for skin-related hygiene practices, which may have affected the skin microbiota.

When compared to skin spacers, the proportion of shared spacers in saliva over time in each subject was highly significant (p < 0.005 in all subjects for SGII and SGI spacers) (Additional file 1: Table S4). In some cases there were more shared spacers between skin and saliva than there were for comparisons of different time points within the skin of the same subject for SGII spacers (44% shared between saliva and skin versus 37% shared in skin for Subject #1; 41% vs 36% in Subject #2; 11% vs 15% for Subject #3; 25% vs 24% for Subject #4) and for SGI spacers (42% shared between saliva and skin versus 39% shared in skin for Subject #1; 30% vs 28% in Subject #2; 16% vs 10% for Subject #3; 37% vs 36% for Subject #4). These data demonstrate a smaller group of shared spacers present on the skin of these subjects than in their saliva, which suggests greater heterogeneity in the skin microbiota.

We also examined spacers shared between different subjects and whether there were any SGI CRISPR spacers shared with SGII spacers. On average, 21.86 ± 1.98% of the SGI spacers were shared between subjects, 20.93 ± 2.34% of the SGII spacers were shared between subjects, while only 0.011 ± 0.004% (p < 0.001) of the SGI and SGII spacers were shared between subjects, indicating that either SGI and SGII spacers likely target different viruses/plasmids, or target different portions of the same viruses/plasmids [37].

### CRISPR locus assembly

**Figure 4:**
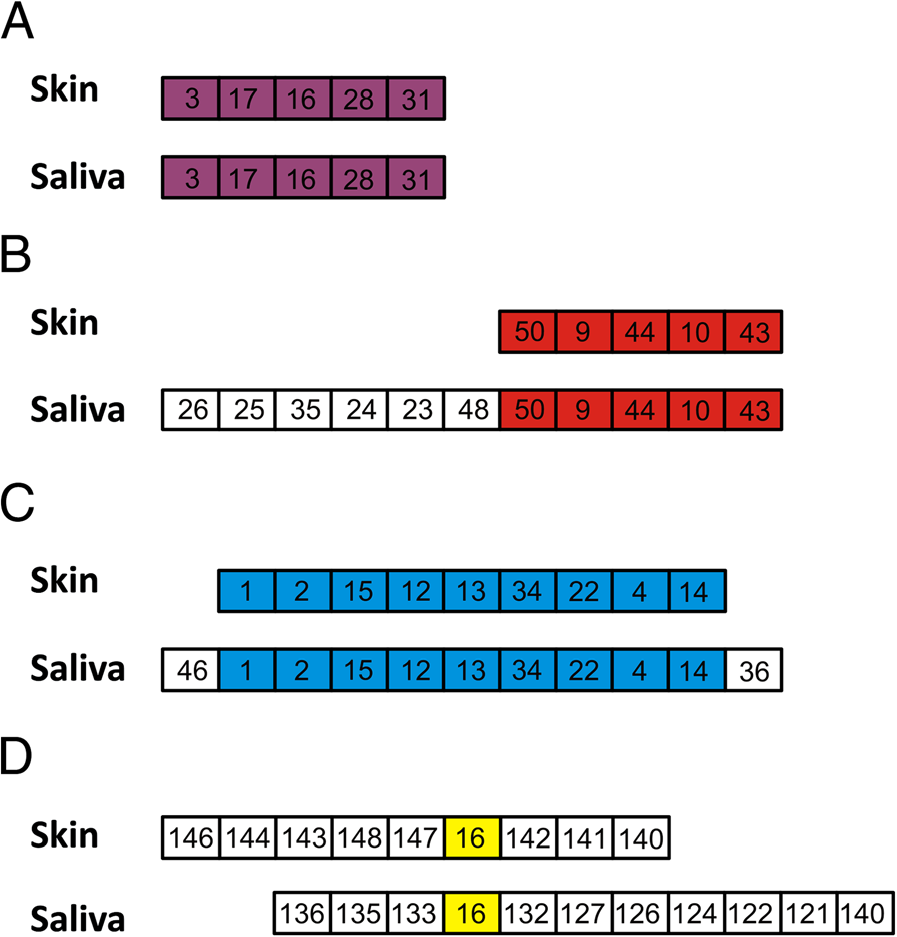
**Assembled CRISPR loci from subject #3 on day 14 in the morning.** Panels **A**-**D** represent different loci that were reconstructed, and shared CRISPR spacers between the loci of the skin and saliva are noted by colored boxes. White boxes represent spacers that were unique to either the skin or saliva. Numbers in the boxes represent the unique identifiers given to each spacer.

Because of the short read lengths of most of the sequences produced in this study, CRISPR loci could not be assembled; however, longer reads sequenced from the day 14 AM sample from subject #3 could be assembled into loci. By using adjacent spacers as scaffolds, we were able to reconstruct 8 different CRISPR loci in the saliva and 28 different CRISPR loci on the skin. Of the 8 loci reconstructed in the saliva, 4 shared at least 1 spacer with loci reproduced on the skin (Figure 4). We identified CRISPR loci that were identical between the skin and saliva (Panel A), that shared a common end (Panel B), that shared a common middle (Panel C), and that only shared a single spacer flanked by spacers not present at the other body site (Panel D). Only a single spacer from any of these 4 loci is identical to any previously sequenced spacers. These data suggest that at least some of the shared spacers on the saliva and skin were derived from loci with shared spacer content and order.

### Analysis of CRISPR spacer variation

**Figure 5:**
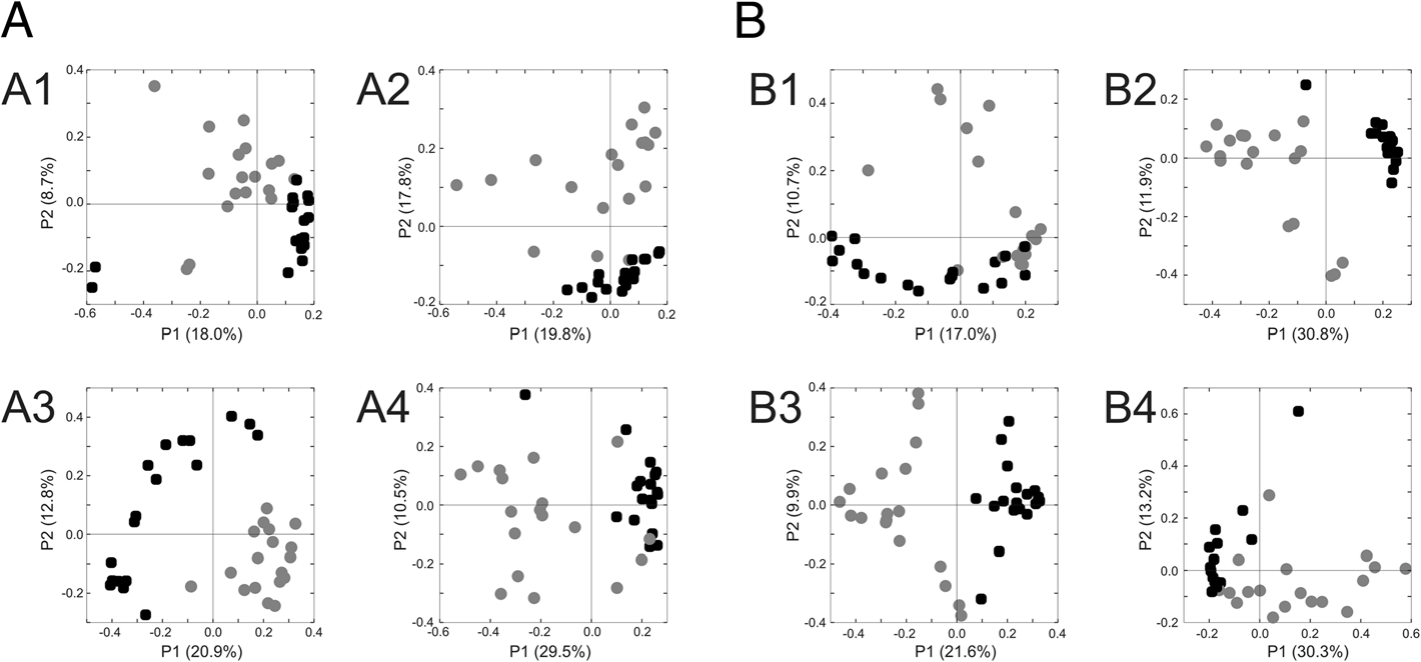
**Principal coordinates analysis of CRISPR spacer groups between skin and saliva.** Beta diversity was determined using Sorensen’s distances. Panel **A** represents SGI CRISPR spacers and Panel **B** represents SGII CRISPR spacers. Subpanel 1 represents Subject#1, Subpanel 2 represents Subject #2, Subpanel 3 represents Subject #3, and Subpanel 4 represents Subject #4. Salivary CRISPRs are represented in black, and skin CRISPRs are represented in gray.

Because there were shared spacers between the saliva and skin of each subject (Figure 2 and Additional file 2: Figure S3), we tested whether the variation present in the spacers in the saliva versus the skin was unique based on environment. Principal coordinates analysis of the CRISPR spacer repertoires examining only the presence/absence of spacers demonstrated that at most time points the biogeographic site was an important determinant of diversity for SGI spacers (Figure 5, panel A) and SGII spacers (Figure 5, panel B). We also used a permutation test [10] to determine whether there was a significant association amongst the spacers by biogeographic site (skin or saliva). Briefly, we tested whether the fraction of shared spacers amongst the skin spacers or amongst the salivary spacers would be greater than for comparisons of spacers on the skin against spacers in saliva. We performed this test by randomly sampling 1,000 spacers from each subject over 10,000 iterations. We found that the estimated fraction of shared spacers over time amongst the salivary spacers was highly significant (p < 0.0001 for each) (Table 1). The estimated fraction of shared spacers amongst the skin spacers of each subject was no greater than for comparisons of skin against saliva, with no significant relationships found. These data indicate that there is a highly significant group of shared SGI and SGII CRISPR spacers present in saliva that is not paralleled on the skin of each subject.

**Table 1:** Estimated shared CRISPR spacers in saliva and skin

	**Saliva**		**Skin**		**Saliva vs skin**
**Subject**	**Percent shared**^**a**^	**p-value**^**b**^	**Percent shared**^**a**^	**p-value**^**b**^	**Percent shared**^**a**^
SGI					
Subject 1	45.91 ± 1.56	**<0.0001**	23.97 ± 1.36	0.9945	29.39 ± 1.51
Subject 2	55.64 ± 1.51	**<0.0001**	27.31 ± 1.41	0.9849	31.78 ± 1.44
Subject 3	23.86 ± 1.37	**<0.0001**	10.27 ± 0.97	0.1584	8.99 ± 0.89
Subject 4	38.60 ± 1.53	**<0.0001**	16.05 ± 1.19	0.6741	16.83 ± 1.17
SGII					
Subject 1	48.13 ± 1.61	**<0.0001**	28.50 ± 1.40	0.9947	34.07 ± 1.56
Subject 2	50.75 ± 1.55	**<0.0001**	21.64 ± 1.31	0.2537	20.50 ± 1.25
Subject 3	35.31 ± 1.51	**<0.0001**	7.64 ± 0.84	0.9827	10.37 ± 0.99
Subject 4	52.52 ± 1.57	**<0.0001**	25.78 ± 1.39	0.9439	28.95 ± 1.41

^a^Based on the mean of 10,000 iterations. 1,000 random spacers were sampled per iteration.

^b^Empirical p-value based on the fraction of times the estimated percent shared spacers for comparisons within skin or saliva exceeds that between skin and saliva. p-values ≤0.05 are represented in bold.

We also examined CRISPR repertoires by collapsing all time points between subjects to determine whether the CRISPR spacers in each environment were a direct reflection of the subject and environment from which they were derived. When considering both the presence of spacers and their abundance in skin and saliva, we found that for most subjects the CRISPR repertoires were significantly subject-specific (Additional file 1: Table S5). We estimated that 94% of the SGII spacers were conserved across the skin and saliva of Subject #1 compared to only 35% when comparing between different subjects (p < 0.0001). Similar results were produced for all subjects for both SGI and SGII CRISPR spacers with the exception of Subject #4 (Additional file 1: Table S5). While the results did not reach statistical significance for Subject#4, the trends in the proportions of intra-subject shared spacers between skin and saliva exceeded inter-subject comparisons substantially (86% vs 57% for SGI spacers and 58% vs 35% for SGII spacers).

### CRISPR spacer matches

**Figure 6:**
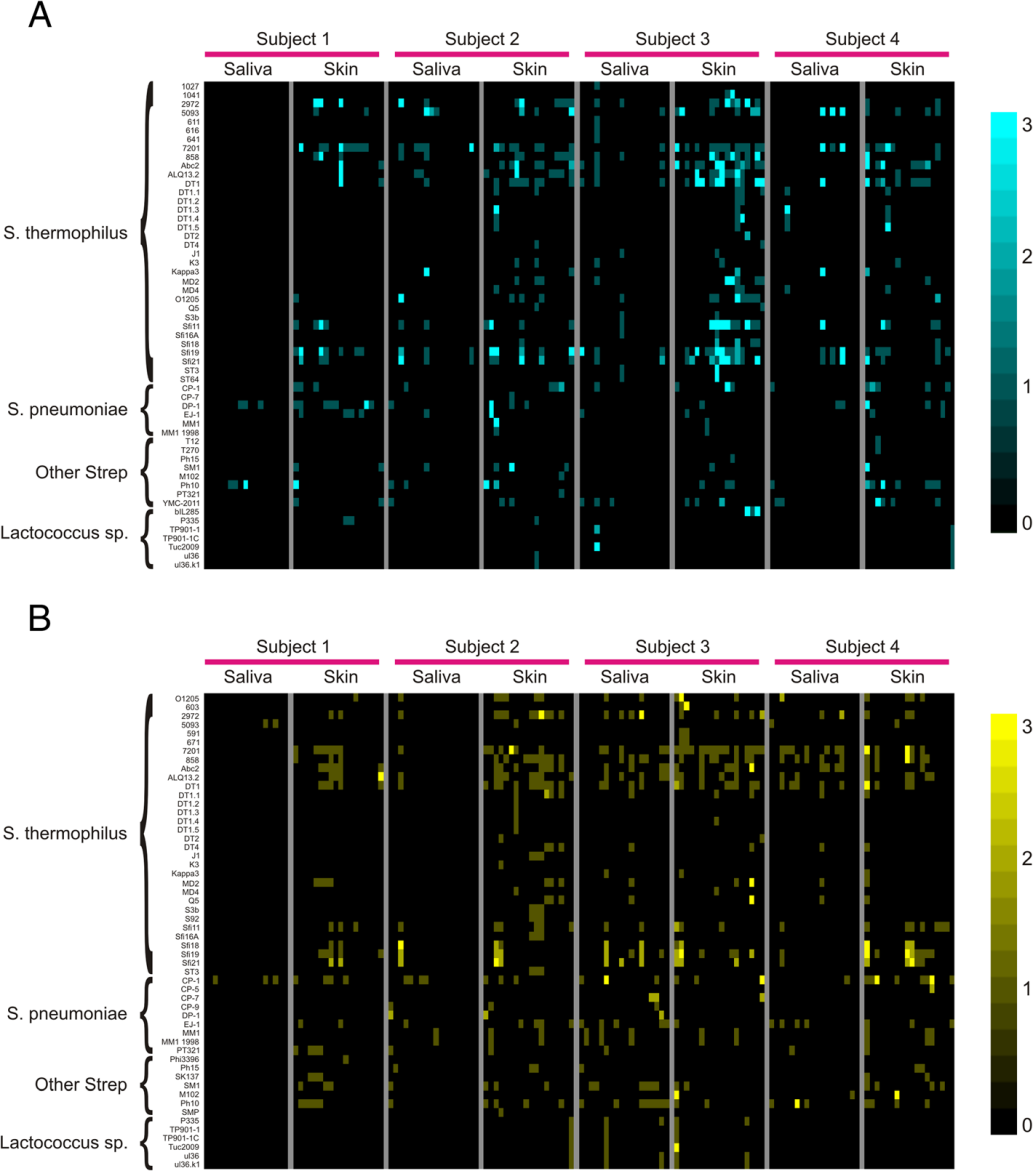
**Heatmaps of CRISPR spacers homologous to bacteriophage in the NCBI Non-redundant database.** Each row represents a unique phage and the columns represent spacers from all individual time points (from left to right) in all subjects. For each column, homologues are only shown for CRISPR spacer groups that were not present at any prior time points in each subject. The subject and sample type are denoted at the top of each heatmap, and the organisms from which the phage were isolated are located on the left. The intensity scale bar is located to the right. Panel **A** – SGII CRISPR spacers and Panel **B** – SGI CRISPR spacers.

We tested whether the spacer repertoires from skin and saliva matched similar viruses (Additional file 2: Figure S6). We found that 8.6% of saliva-derived and 25.3% of skin-derived SGII spacers were homologous to streptococcal viruses in the NCBI Non-redundant (NR) database, and 6.9% of saliva-derived and 15.3% of skin-derived SGI spacers were homologous to streptococcal viruses. Comparatively, only 4.5% of saliva-derived and 6.5% of skin-derived SGII spacers were homologous to streptococcal plasmids, and 0.3% of saliva-derived and 0.9% of skin-derived SGI spacers were homologous to streptococcal plasmids. In all cases, the proportion of skin-derived spacers with homologues in the NR database was significantly (p ≤ 0.005) greater than that for saliva-derived spacers. We created heatmaps of the spacer homologues across all time points for both saliva and skin, where only spacers that were newly identified at each time point were included. For many of the viral homologues, newly identified spacers targeted different portions of the same viruses over time for both SGI spacers (Figure 6, panel A) and SGII spacers (Figure 6, panel B), suggesting that the bacteria harboring these CRISPR spacers either acquired additional resistance motifs against the same or similar viruses or that new bacterial strains harboring spacers targeting these viruses entered the community. Most of the viruses that matched CRISPR spacers in this study were previously identified in *S. thermophilus* and *S. pneumoniae*. We also noted that 38.7 ± 0.09% of the SGII and 40.4 ± 0.11% of the SGI spacers on the skin matched the same viruses as those spacers identified in saliva. Approximately 53.3 ± 1.2% of the SGII and 40.4 ± 3.2% of the SGI CRISPR spacers from different subjects matched the same viruses. A few of the spacers matched viruses found in species of *Lactococcus*, which are closely related to *Streptococcus*.

**Figure 7:**
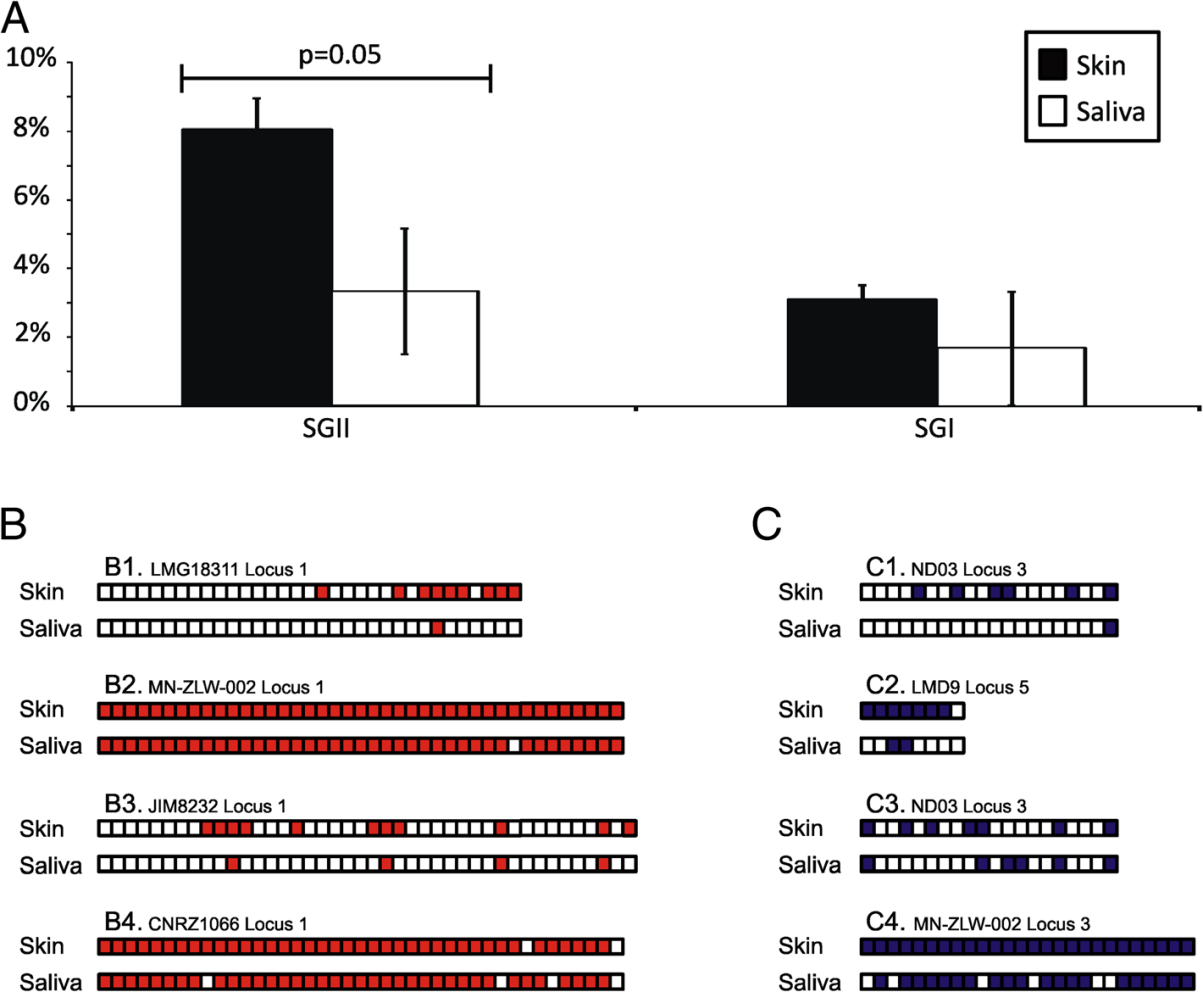
**Percentage of CRISPR spacers (±standard deviation) that match spacers from previously identified CRISPR loci in the CRISPR Database (Panel A), and profiles of the spacer matches to specific CRISPR loci from different strains of *S. thermophilus *for SGII (Panel B) and SGI (Panel C) spacers.** In panels **B** and **C**, each box represents a spacer in a CRISPR locus in the CRISPR Database, and colored boxes represent spacers that also were present in this study. White boxes represent spacers that were not identified in this study. In each subpanel, the colored boxes from the top locus represent spacers that were matched by skin-derived spacers, and the bottom box represents spacers that were matched by saliva-derived spacers.

We also compared the CRISPR spacers from the skin and saliva of all subjects to determine whether there might be spacers in our cohort that matched those identified in previously sequenced CRISPR loci. We found that 2-8% of the CRISPR spacers were also found in loci from the CRISPR database [38], with the number of skin spacers found in the database generally exceeding salivary spacers (Figure 7, Panel A). While there were spacers identified that matched loci from many different streptococcal species, the majority of the loci belonged to *S. thermophilus*. For example, many of the SGII 3’ spacers from CRISPR Locus 1 of *S. thermophilus* LMG18311 were identified on the skin of subject #1, but only 1 of those spacers was identified in the saliva (Figure 7, Panel B1). All of the SGII spacers in Locus 1 of *S. thermophilus* MN-ZLW-002 were identified on the skin of subject #2, but 1 was missing in the saliva of that subject (Panel B2). Similar patterns of shared spacers were found in subjects #3 and #4 (Panels B3-B4). SGI spacers also matched spacers from various *S. thermophilus* loci (Panels C1-C4). These data suggest that loci similar to those isolated from *S. thermophilus* were sampled on both the skin and saliva of our study subjects.

**Figure 8:**
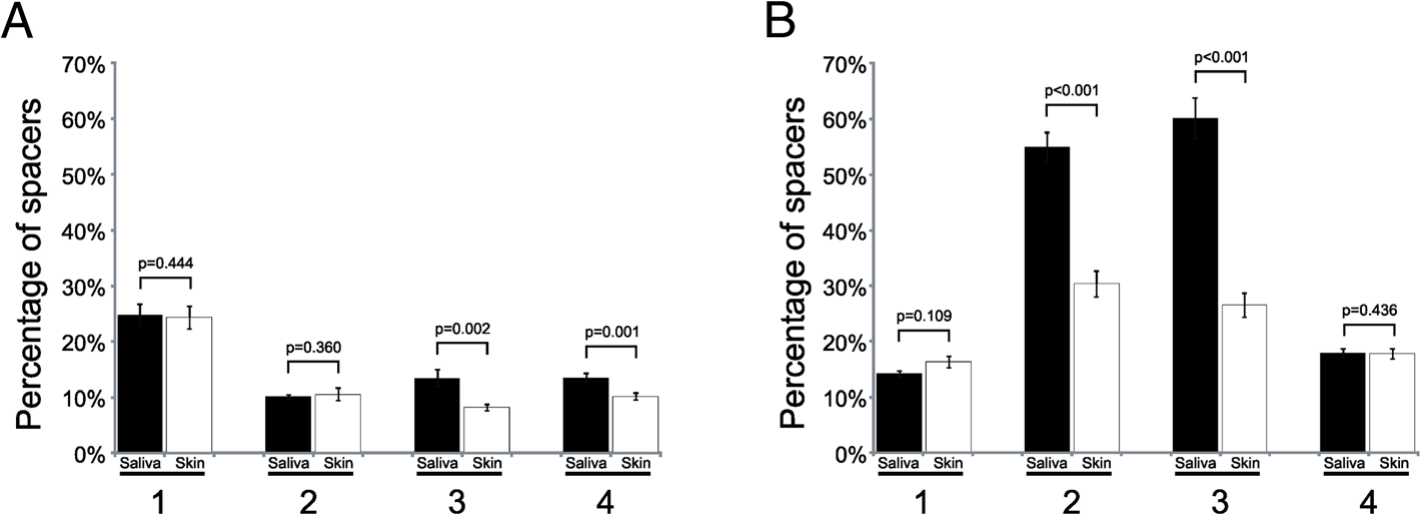
**Percentage of SGI (Panel A) and SGII (Panel B) CRISPR spacers matching virome reads from the saliva of 21 human subjects **[10]**.** The Y-axis shows the mean percentage of the CRISPR spacers from all time points combined that matched virome reads from the cohort of 21 subjects. The X-axis represents the saliva- and skin-derived spacers for each subject. Standard error bars are represented above each bar, and the p-value is demonstrated above each error bar. Subjects 1 through 4 are shown consecutively from left to right on the X-axis.

To determine whether skin-derived CRISPR spacers matched viruses present in the saliva, we sequenced the viromes present in each of our subjects’ saliva on Day 1 and Week 8. Similar to our previous studies [14], the proportion of CRISPR spacers matching virome reads was relatively low. When examining the pooled reads from all subjects, we found that between 0.0% and 1.0% of the CRISPR spacers in each subject matched virome reads for SGI spacers and SGII spacers (Additional file 2: Figure S7). When we tested the skin- and saliva-derived spacers against a larger database of salivary viromes from a cohort 21 human subjects [10], we found that a high number of salivary- and skin-derived spacers matched salivary virome reads (range from 14 to 60% for SGII spacers and 10 to 24% for SGI spacers). The proportion of spacers matching salivary viruses was significantly (p ≤ 0.002) higher for saliva-derived spacers than for skin-derived spacers for Subjects #3 and #4 for SGII spacers, but not Subjects #1 and #2. There also were a significantly higher proportion of SGI saliva-derived spacers that matched salivary viruses in Subjects #2 and #3, but not Subjects #1 and #4 (Figure 8).

We also tested whether there were matches to spacers found in previously sequences metagenomes recovered from the human oral cavity [39], the gastrointestinal tract [40], and human skin [41]. We found that a significantly higher percentage of SGII (3-4%) and SGI (4-5%) spacer sequences were found in oral metagenomes than the 1-2% of SGII and SGI found in the gut and the <1% found on the skin (p < 0.02) (Additional file 2: Figure S8, Panels A and B). While the significant difference between spacers shared between oral and gut metagenomes is consistent with a prior study [14], the lack of spacers found on skin metagenomes may be secondary to the different surfaces sampled between studies, where the retroauricular surface was sampled to produce the skin metagenomes compared to the volar forearm surface used in this study.

### Spacer rate change

Little is known about the rate at which spacers are acquired for bacteria in human ecosystems. Due to our repeat motif based amplification approach, we were unable to discern between newly acquired spacers in existing bacteria and those that may be newly identified because of new bacteria entering the environment. We could, however, compare the estimated rates of newly identified spacers between skin and saliva. To estimate the number of spacers at each time point, we corrected for the probability that any spacer present at a given time point might not be observed due to variations in sampling. For SGII spacers the estimated rate of newly identified spacers per hour for skin exceeded that for saliva in all subjects, and was significant (p < 0.05) for 3 of the 4 subjects (Additional file 2: Figure S9, Panel A). Similar trends were not observed for SGI spacers (Additional file 2: Figure S9, Panel B), where only in subject #2 did the estimated rate for skin significantly exceed saliva. The overall rate per hour of newly identified SGII spacers was significantly higher for skin (15.8 ± 1.7) than for saliva (7.6 ± 1.2; p < 0.001), while it was similar for skin (16.9 ± 1.8) and saliva (16.3 ± 2.6; p = 0.422) for SGI spacers.

### Bacterial community variation

Because many of the SGI and SGII CRISPR spacers were subject specific and shared between skin and saliva, we also characterized the bacterial communities in each subject to ensure that the microbiota of each body site were distinct. We sequenced a total of 2,020,553 reads from the V3 region of 16S rRNA, for an average of 21,047 reads per time point and sample type for all subjects over the 8-week study period. We performed principal coordinates analysis for the bacterial communities to determine whether the variation in these communities may be subject specific and reflective of the body site from which they were derived, as had been demonstrated for SGI and SGII CRISPRs (Figure 5). The majority of the variation observed between skin and saliva was on the x-axis, which accounted for 66% of the observed variation (Additional file 2: Figure S10). The bacterial communities from both saliva and skin appeared to be highly specific to the body site examined, but not subject specific. We quantified the proportion of shared OTUs (Operational Taxonomic Units) within and between the skin and saliva of each subject, and found that there was a significant proportion conserved in the saliva of each subject (p ≤ 0.05; Additional file 1: Table S6). While only Subjects #1, #3, and #4 had significant proportions of shared OTUs (p ≤ 0.05) on the skin, the proportion shared on the skin of Subject #2 substantially exceeded those shared between the saliva and skin (62% vs. 36%; p = 0.24). There also was a greater abundance of streptococci in the saliva than on the skin of each subject (mean 29.8 ± 2.2% vs 5.8 ± 1.3%; p < 0.001) (Additional file 2: Figure S11, Panels A and B). These data indicate that while there were discernible differences between the bacterial communities and CRISPR spacer profiles of the skin and saliva for each subject, only the CRISPR spacer profiles were subject specific.

## Discussion

The study of viruses inhabiting body surfaces is still in its relative infancy. Because little biomass can be obtained non-invasively from the skin, viral communities on this surface remain relatively poorly characterized. Others have begun to characterize some of the features of the viruses in this broad ecosystem [23], yet their analysis has not identify many viruses of bacteria. Because of the abundance of bacteria inhabiting human skin, the skin might be expected to be inhabited by many bacteriophage, as has previously been demonstrated for the human oral cavity [1,2] and gut [4,7]. We sampled CRISPRs because their profiles may shed light into the diverse features and types of viruses to which streptococci on the skin might encounter in nature, and might be contrasted with viruses found in saliva. While we found many spacers on skin that matched those of saliva, many may belong to loci that have been either vertically or horizontally inherited; thus, the similarities between skin and salivary CRISPR spacer profiles may not reflect independent viral encounters. It does represent an intriguing possibility that bacteria on the skin and saliva encounter similar viruses, but this study was not designed to demonstrate that phenomenon.

While there were relatively few CRISPR spacers that matched salivary viruses from the subjects in this study, there were many that matched viruses from a larger cohort of different subjects [10]. We previously demonstrated that CRISPR spacer/virome matches generally are not subject specific [14], and we believe that this phenomenon may be due to heterogenous representation of viruses between different subjects. For example, similar viruses may be present in both subjects, but in one subject one virus may be highly abundant at the time of sampling, while in another subject it is not. Therefore, by comparing CRISPR spacers to viromes from multiple subjects, we may identify matches to viruses that are of otherwise too low an abundance to be identified in our cohort.

The repeat-based amplification technique used in this study was not without limitations, including that we could not ascribe most spacers to bacterial species or CRISPR loci [15]. Additionally, CRISPR spacers could have been amplified from loci that are similar but not identical to SGII and SGI CRISPR repeat motifs [42]. By removing any altered CRISPR repeat motifs from the analysis, we also could limit the potential effect of amplifying non-streptococcal species that might bear similar repeat motifs. We believe that much of the similarity observed in our analysis of SGI and SGII CRISPRs between the skin and saliva was due to the presence of different streptococcal species harboring these repeat motifs on both body sites, as the vast majority of homologous sequences found to our CRISPR spacers were from streptococcal phage, plasmids, and genomes. We attempted to find and include spacer sequences from CRISPR repeat motifs not known to be present in *Streptococcus*, including repeat motifs found in species of *Gemella*, *Veillonella*, *Leptotrichia*, and *Kingella*, but their presence was not uniform on the skin (data not shown).

Because of the error rate of Ion Torrent sequencing [36], we took additional precautions to reduce sequencing error biases in our analysis of CRISPR spacers. Each CRISPR-bearing read was trimmed according to quality scores, and was removed if it had significant homopolymer tracts. We specifically removed any CRISPR-bearing reads from the analysis that did not match the known consensus repeat motifs, as those reads were more likely to contain sequencing errors. The combination of these techniques reduced the error rate from approximately 1% to an estimated 0.001 and 0.002% for SGI and SGII CRISPR spacers, respectively. Our previous studies of CRISPR repertoires in humans had been performed using conventional Sanger sequencing, however we now have extended our analysis using next-generation sequencing techniques. The primary benefit of the current technique was that we were able to achieve greater sampling depth, which allowed for more robust comparisons of skin and salivary CRISPRs with fewer unsampled spacers.

Our data on shared CRISPR spacers between skin and saliva revealed several qualities about CRISPRs on human body surfaces: 1) fewer spacers were shared between subjects than within subjects, suggesting that CRISPR repertoires were individual specific, 2) the substantial persistence of spacers, suggesting that the bacteria harboring them were conserved over the time period studied, and 3) the level of shared spacers between skin and saliva in individual subjects (Figure 1 and Additional file 2: Figure S2), which raises the possibility that skin-derived bacteria may have encountered viruses with similar sequences to those in the mouth. While it is possible that some of the spacers were acquired through independent means [10], the substantial levels of shared spacers between skin and saliva suggests some vertical or horizontal acquisitions. Despite our inability to reconstruct many CRISPR loci using this short-read technology, our finding that many spacers from previously sequenced *S. thermophilus* isolates were present in this cohort suggests that those loci may be present in this study with their spacer content and order intact. Because the location of the CRISPR loci in our subjects was variable, we were unable investigate them robustly by PCR amplification using their flanking regions, followed by Sanger sequencing.

We initially hypothesized that there would be large groups of spacers specific to saliva and specific to skin that would be unique to each body surface. We found that there was a group of salivary spacers that were highly persistent over the 8-week study period, but the same trend was not true for skin spacers (Table 1). We believe that the lower level of spacer persistence on skin may be secondary to increased heterogeneity in skin bacterial populations over time. We analyzed the bacterial populations using 16S rRNA specifically to substantiate that there were differences between skin and salivary microbiota in these subjects, as the substantial levels of shared CRISPR spacers between the body sites in such a large dataset were unexpected. The segment of 16S rRNA sequenced was not sufficient to differentiate different streptococci at the species level, but was sufficient to discern differences between the microbiota of each body site.

## Conclusions

We aimed to characterize streptococcal CRISPR spacer profiles of distinct human biogeographic sites to determine whether CRISPR spacers were highly conserved over time. We found that there were robust repertoires of spacers from both sites, but neither profiles were fully ecologically distinct. There were abundant shared spacers between the skin and saliva of all 4 subjects (Figure 1), suggesting vertical or horizontal acquisition of CRISPR loci among the streptococci inhabiting these body sites. The significant group of temporally conserved spacers in saliva was much larger than that found on skin (Table 1), which might reflect a higher diversity of cutaneous bacterial strains. While many of the CRISPR spacers identified in saliva matched concurrent viruses in saliva, the relatively high proportion of skin-derived spacers matching salivary viruses warrants further study to determine whether streptococci on the skin may encounter viruses with similar sequences to those in the mouth.

## Methods

### Human subjects

This full study including the enrollment of human subjects and the consent procedure was approved by the University of California, San Diego and the Western University institutional review boards. Each subject donated saliva samples and skin swabs three times daily at various time points over an 8-week period (Day 1 AM, Noon, PM; Day 2 AM, Noon, PM; Day 4 AM, Noon, PM; Day 14 AM, Noon, PM; Day 28 AM, Noon, PM; Week 8 AM, Noon, PM). Prior to sample collection, each subject completed a survey self-reporting his or her oral health and any other pre-existing medical conditions that could result in substantial immunosuppression, and reported themselves to be in good overall cutaneous and periodontal health. Exclusion criteria also included antibiotic administration during the 12 months prior to the beginning of the study. Each subject provided a minimum of 3 ml of non-stimulated saliva at all time points, and a skin swab from the volar surface of their forearm. The same volar surface from the same arm was used for each subject throughout all time points sampled. Samples from skin were collected on a swab soaked in a solution of 0.15 M NaCl and 0.1% Tween 20 and were resuspended in PBS [31]. Saliva and skin samples were frozen at -80°C prior to use in this study. All AM time points were collected prior to meals or oral hygiene practices, the noon time point was collected prior to lunch, and the PM time point was collected prior to dinner. The study was not controlled for cutaneous hygiene practices.

### Amplification and binning of streptococcal CRISPR spacers

From each subject, genomic DNA was prepared from saliva and skin using Qiagen QIAamp DNA MINI kit (Qiagen, Valencia, CA). Each sample was subjected to a bead beating step prior to nucleic acid extraction using Lysing Matrix-B (MP Bio, Santa Ana, CA). SGI and SGII CRISPR primers were designed based on their specificity to the CRISPR repeat motifs present in *S. gordonii* str. Challis substr. CH1 and *S. mutans* UA159, and included barcode sequences (Additional file 1: Table S1) [14]. Each primer was used to amplify CRISPRs from saliva and skin-derived DNA by PCR. Reaction conditions included 45 μl Platinum High Fidelity Supermix (Life Technologies, Grand Island, NY), 1 μl of each of the forward and reverse primer (20 pmol each), and 3 μl salivary or skin-derived DNA template. The cycling parameters were 3 minutes initial denaturation at 95°C, followed by 30 cycles of denaturation (60 seconds at 95°C), annealing (60 seconds), and extension (5 minutes at 72°C), followed by a final extension (10 minutes at 72°C). CRISPR amplicons were gel extracted using the Qiagen MinElute Kit (Qiagen, Valencia, CA) including buffer QG and further purified using Ampure beads (Beckman-Coulter, Brea, CA). Molar equivalents were determined from each product using an Agilent Bioanalyzer HS DNA Kit (Agilent, Santa Clara, CA), and each were pooled into molar equivalents. Resulting pools were sequenced on 314 chips using an Ion Torrent Personal Genome Machine (PGM) according to manufacturer’s instructions (Life Technologies, Grand Island, NY) [36]. Barcoded sequences then were binned according to 100% matching barcodes. Each read was trimmed according to modified Phred scores of 0.5, and low complexity reads (where >25% of the length were due to homopolymer tracts) and reads with ambiguous characters were removed prior to further analysis using CLC Genomics Workbench 4.65 (CLC bio USA, Cambridge, MA). Only those reads that had 100% matching sequences to both the 5’ and the 3’ end of the CRISPR repeat motifs were used for further evaluation. Spacers were defined as any nucleotide sequences (length ≥20) in between repeat motifs. Spacers then were grouped according to their trinucleotide content, as previously described [10]. Briefly, the trinucleotide content was compiled for all spacers and added to a database. For each spacer sequence, the difference in trinucleotide content was compared between all possible spacer pairs. The sum of the differences for all spacer pairs then was determined for all sequences, and then spacers were binned together if their differences were less than the standard deviation from the mean overall difference.

For each subject evaluated, a database of spacer groups was generated, and databases were compared to determine shared spacer groups and to create heatmaps using Java Treeview [43]. Spacer heat matrices were created using Microsoft Excel 2007 (Microsoft Corp., Redman, WA). Beta diversity was determined using binary Sorensen distances, and was used as input for principal coordinates analysis using Qiime [44]. Spacers from each subject were subjected to BLASTN [34] analysis based on the NCBI Non-redundant database. Hits were considered significant based on bit scores ≥45, which roughly correlates to 2 nucleotide differences over the length of a 30 nucleotide spacer. The number of blast homologues then were normalized for each subject, and heatmaps were created using Java Treeview [43]. Spacers also were queried against the loci present in the CRISPR Database [38] or other specified metagenomic datasets, and only spacers that were identical or had a single mismatch over the entire length of the spacer were considered matches. CRISPR spacers for each subject were used to search a database of the virome reads for matches from all viromes combined, and the number of spacer matches per virome read was used to create heatmaps. The heatmaps were normalized by the total number of spacer matches per virome read, and were generated using Java Treeview [43]. Rarefaction analysis was performed based on spacer group richness estimates of 10,000 iterations using EcoSim [45]. CRISPR loci were reassembled from reads that had a minimum of 2 full spacer sequences flanked by full-length repeat motifs. Each locus was reassembled based on matching adjacent spacers, in which reads were only assembled into loci if their adjacent spacers were present in the same combination in at least 75% of the reads assessed.

### Isolation and analysis of viromes

Saliva from human subjects was filtered sequentially through 0.45 μ and 0.2 μ filters to remove cellular debris, and the remaining fraction purified on a cesium chloride gradient as previously described [8]. Only the fraction at the density of most known viruses [46] was retained; it was then further purified on Amicon YM-100 protein purification columns (Millipore, Inc., Bellerica, MA), and treated with DNASE I, followed by lysis and DNA purification using Qiagen UltraSens virus kit (Qiagen, Valencia, CA). Resulting DNA was amplified using GenomiPhi V2 MDA amplification (GE Healthcare, Pittsburgh, PA), fragmented to roughly 100 to 200 bp using a Bioruptor (Diagenode, Denville, NJ), constructed into libraries using the Ion Plus Fragment Library Kit according to manufacturer’s instructions, and sequenced using 316 chips on an Ion Torrent PGM (Life Technologies, Grand Island, NY) [36] producing an average read length of approximately 100 bp for each sample. Each read was trimmed according to modified Phred scores of 0.5 using CLC Genomics Workbench 4.65 (CLC bio USA, Cambridge, MA), and low complexity reads (where >25% of the length were due to homopolymer tracts) were removed prior to further analysis. Any remaining reads with substantial length variation (<50 nucleotides or >200 nucleotides) or reads with ambiguous characters were removed from the analysis. To ensure that the viral communities were properly separated from potential contaminating cellular elements, we screened each virome against the RDP 16S ribosomal RNA database [47] and the RefSeq human database available at NCBI (ftp://ftp.ncbi.nlm.nih.gov/refseq/H_sapiens/). CRISPR spacer/virome read matches were defined as virome sequences that were identical or had a single nucleotide mismatch when compared to the CRISPR spacer sequences.

### Analysis of 16S rRNA

We amplified the bacterial 16S rRNA V3 hypervariable region using the forward primer 341 F (CCTACGGGAGGCAGCAG) fused with the Ion Torrent Adaptor A sequence and one of 23 unique 10 base pair barcodes, and reverse primer 514R (ATTACCGCGGCTGCTGG) fused with the Ion Torrent Adaptor P1 from the skin and salivary DNA of each subject [48]. PCR reactions were performed using Platinum PCR SuperMix (Invitrogen, Carlsbad, CA) with the following cycling parameters: 94°C for 10 minutes, followed by 30 cycles of 94°C for 30 seconds, 53°C for 30 seconds, 72°C for 30 seconds, and a final elongation step of 72°C for 10 minutes. Resulting amplicons were purified on a 2% agarose gel stained with SYBR Safe (Invitrogen, Carlsbad, CA) using the MinElute PCR Purification kit (Qiagen, Valencia, CA). Amplicons were further purified with Ampure beads (Beckman-Coulter, Brea, CA), and molar equivalents were determined for each sample using a Bioanalyzer 2100 HS DNA Kit (Agilent Technologies, Santa Clara, CA). Samples were pooled into equimolar proportions and sequenced on 314 chips using an Ion Torrent PGM according to manufacturer’s instructions (Life Technologies, Grand Island, NY) [36]. Resulting sequence reads were removed from the analysis if they were <130 nt, had any barcode or primer errors, contained any ambiguous characters, or contained any stretch of >6 homopolymers. Sequences were assigned to their respective samples based on their 10-nt barcode sequence, and were analyzed further using the Qiime pipeline [45]. Briefly, representative OTUs from each set were chosen at a minimum sequence identity of 97% using UClust [49] and aligned using PyNast [50] against the Greengenes database [51]. Multiple alignments then were used to create phylogenies using FastTree [52], and taxonomy was assigned to each OTU using the RDP classifier [53,54]. Principal coordinates analysis was performed based on Beta Diversity using weighted Unifrac distances [55].

### Statistical analysis

To assess whether spacer groups had significant overlap between the skin and saliva for each subject, we performed a permutation test. We simulated the distribution of the fraction of shared spacer groups from 2 different time points within individual subjects that were randomly chosen across all time points. For each set, we computed the summed fraction of shared spacer groups comparing randomly chosen skin spacers with randomly chosen salivary spacers, and from these computed an empirical null distribution of statistics. The fraction computed in each of 10,000 iterations resulted from the random sampling of 1000 spacer groups. The standard deviation was computed from the percentage of shared spacer groups over the 10,000 iterations. The simulated statistics for the skin and saliva in each subject were referred to the null distribution comparing skin and salivary spacers, and the *p* value was computed as the fraction of times the simulated statistic for the each exceeded the null distribution. The same technique was utilized for 16S rRNA OTUs and to test the proportions of shared spacers in each subject by time of day.

To determine a relative rate at which new spacers were identified in each subject and sample type, we estimated the number of shared spacers between two samples (observed at different times). A naive estimate that simply computes the number of spacers observed at both times or each time exclusively to estimate these quantities does not take into account statistical variation in spacer content due to sampling depth, or the chance that a spacer will not be observed due to Poisson sampling. To estimate this bias, n10, n01 and n11 respectively denote the number of spacer groups present at the first sampling time point and not the second, the second but not the first, and both samples. By using the empirical estimates of these quantities, we could correct for any underestimates from using the observed numbers of spacer groups. We therefore used a statistical model to correct for this bias and estimate the rate of change between spacer populations. To estimate each of these three quantities, we used statistics s10, s01, s11 representing the observed numbers of spacer groups in each category, but each was necessarily an underestimate of n10, n01 and n11. p and q denote the probabilities of seeing a spacer group if it is present at time 0 or time 1. The expectation of each can be calculated as: E(s01) = (((1-q)*n01) + ((1-p)*(q*n11))), E(s10) = (((1-p)*n10) + ((1-q)*(p* n11)), and E(s11) = (p*q*n11), where p = 1/N sum_i e^{-lambda_i} for sample 1 and q = 1/N sum_i e^{-lambda_i} for sample 2, where lambda_i is the depth that spacer group i is sampled. These estimates were used to determine the proportion of spacers shared between consecutive time points for each subject and sample type.

Comparisons of the mean percentages of shared spacers and standard error rates in different subjects or between the skin and saliva of each subject were performed using Microsoft Excel 2007 (Microsoft Corp., Redman, WA). Statistical significance was determined by two-tail t-test for comparison of means, and by single factor ANOVA when comparing the proportions of shared spacers between SGI and SGII CRISPR spacer types.

### Availability of supporting data

All sequences are available for download in the MG-RAST database (metagenomics.anl.gov/) under the project ‘CRISPR Skin Saliva Project’. Virome sequences are available under consecutive individual accession numbers 4513846.3 to 4513853.3, and 16S rRNA sequences are available under consecutive individual accession numbers 4514730.3 to 4514825.3.

## Competing interests

The authors declare that they have no competing interests.

## Authors’ contributions

Conceived and designed experiments: DTP. Performed the experiments: RR-S, MN, ML, and SRA. Analyzed the data: DTP, JS, and SRA. Collected specimens: TB and DTP. Wrote the manuscript: DTP. All authors read and approved the final manuscript.

## Supplementary Material

Additional file 1: Table S1 CRISPR repeat motifs and primers used in this study. **Table S2.** Presence of SGI and SGII CRISPR repeat motifs in different species. **Table S3.** Reads and spacer counts from the skin and saliva of all subjects. **Table S4.** Mean percentages (±standard error) of shared spacers in the skin and saliva of all subjects for SGI and SGII spacers. Significance values were determined by two-tailed t-tests. **Table S5.** Estimated percentages of shared spacers on the skin and saliva of each subject. **Table S6.** Estimated proportions of shared OTUs on the skin and saliva of each subject. Click here for file

Additional file 2: Figure S1 Rarefaction analysis of CRISPR spacer groups in the saliva and on the skin of all subjects. **Figure S2.** Heatmaps of SGII CRISPR spacer groups in all subjects. **Figure S3.** SGII CRISPR spacer group heat matrices from all subjects. **Figure S4.** Conservation of CRISPR spacer content by time of day sampled. **Figure S5.** Conservation of CRISPR spacer content by time of day sampled. **Figure S6.** Percentage of SGI (Panel **A**) and SGII (Panel **B**) CRISPR spacers with homologues in the NCBI NR database. **Figure S7.** Percentage of SGI (Panel **A**) and SGII (Panel **B**) CRISPR spacers matching virome reads from the subjects in this study. **Figure S8.** Bar graphs representing the percentage of CRISPR spacers (±standard deviation) with matches in human skin, oral, and gut-derived metagenomes. **Figure S9.** Relative rates of newly identified CRISPR spacers in skin and saliva of all subjects. **Figure S10.** Principal coordinates analysis of bacterial OTUs based on 16S rRNA sequences for the skin and saliva of all subjects. **Figure S11.** Percentage of taxonomic assignments from the Genus *Streptococcus* in all subjects for saliva and skin. Click here for file
