# Supplementary material for: Conservation of streptococcal CRISPRs on human skin and saliva
Source: BMC Microbiol. 2014 Jun 6;14:146. doi: 10.1186/1471-2180-14-146 (PMC4063239; doi:10.1186/1471-2180-14-146)
Supplement: Additional file 1: Table S1 — CRISPR repeat motifs and primers used in this study. Table S2. Presence of SGI and SGII CRISPR repeat motifs in different species. Table S3. Reads and spacer counts from the skin and saliva of all subjects. Table S4. Mean percentages (±standard error) of shared spacers in the skin and saliva of all subjects for SGI and SGII spacers. Significance values were determined by two-tailed t-tests. Table S5. Estimated percentages of shared spacers on the skin and saliva of each subject. Table S6. Estimated proportions of shared OTUs on the skin and saliva of each subject. [file 1471-2180-14-146-S1.pdf]

Supplemental Table 1. CRISPR Repeat Motifs and Primers

Name		
**Streptococcus (SGII)**[a]	Consensus	GTTGTACAGTTACTTAAATCTTGAGAGTACAAAAAC
	Forward	CCATCTCATCCCTGCGTGTCTCCGACTCAGXXXXXXXXXXCAGTTACTTAAATCTTGAGAG
	Reverse	CCTCTCTATGGGCAGTCGGTGATAGATTTAAGTAACTGTACAAC
**Streptococcus (SGI)**[b]	Consensus	GTTTTGGAACCATTCGAAACAACACAGCTCTAAAAC
	Forward	CCATCTCATCCCTGCGTGTCTCCGACTCAGXXXXXXXXXXCGTTTACGGTATTGAAAC
	Reverse	CCTCTCTATGGGCAGTCGGTGATGGTAAGCACTGTTGCAAC

[a]Identified from Streptococcus gordonii

[b]Identified from Streptococcus mutans

[c]X’s represent the location of the barcode sequence

Supplemental Table 2. CRISPR Repeat Motifs in different organisms

CRISPR type	SGII	SGI
**Oral streptococci**	*S. gordonii*<br>*S. thermophilus*<br>*S. gallolyticus*<br>*S. anginosus*<br>*S. intermedius*<br>*S. salivarius*<br>*S. suis*<br>*S. pasteurianus*<br>*S. macedonicus*	*S. mutans*<br>*S. thermophilus*<br>*S. gallolyticus*<br>*S. anginosus*<br>*S. infantarius*<br>*S. dysgalactiae*<br>*S. equi*
**Cutaneous streptococci**		*S. pyogenes*[a]<br>*S. agalactiae*
**Other**		*Listeria monocytogenes*[b]<br>*Listeria innocua*[b]

[a]Also found in the oral cavity of about 12% of healthy individuals

[b]Not normal flora in humans

Supplemental Table 3. Spacer sequences from all subjects

		Number of Reads	Number of Spacers	Number of Spacer Groups
SGI Spacers				
Subject 1				
	Saliva	561,973	331,082	822
	Skin	546,623	266,008	1,184
	Combined[a]	1,108,596	579,090	1,637
Subject 2				
	Saliva	380,140	235,840	637
	Skin	423,844	246,840	1,133
	Combined[a]	803,984	329,809	1,261
Subject 3				
	Saliva	558,853	333,366	1,429
	Skin	523,414	318,021	1,098
	Combined[a]	1,082,267	651,387	1,967
Subject 4				
	Saliva	398,552	236,487	624
	Skin	404,673	245,268	1,306
	Combined[a]	803,225	481,755	1,584
SGII Spacers				
Subject 1				
	Saliva	483,220	208,115	273
	Skin	553,237	268,116	1,618
	Combined[a]	1,036,457	476,231	1,685
Subject 2				
	Saliva	756,487	321,064	1,510
	Skin	550,527	233,815	1,014
	Combined[a]	1,307,014	451,734	1,171
Subject 3				
	Saliva	480,322	259,852	531
	Skin	344,394	177,200	944
	Combined[a]	824,716	436,952	1,179
Subject 4				
	Saliva	467,726	243,962	498
	Skin	357,633	165,901	997
	Combined[a]	824,359	409,863	1,088

[a]Determining by combining all spacers from all time points prior to grouping

Supplemental Table 4. Percentage of Shared Spacers

	Mean percentage (± Standard Error) of shared spacers across all time points			P values	
	**Saliva**	**Skin**	**Shared (in saliva and skin)**	**Saliva vs skin**	**Skin vs shared (in saliva and skin)[a]**
SGI					
Subject 1	61.17 ± 2.30	37.55 ± 1.11	43.59 ± 1.03	<0.0001	<0.0001
Subject 2	62.05 ± 0.89	36.24 ± 1.03	41.11 ± 0.63	<0.0001	<0.0001
Subject 3	36.15 ± 1.45	15.01 ± 0.81	10.91 ± 0.39	<0.0001	<0.0001
Subject 4	49.40 ± 1.30	24.36 ± 0.80	24.71 ± 0.75	<0.0001	0.3743
SGII					
Subject 1	44.87 ± 1.49	39.10 ± 1.54	41.79 ± 1.05	0.0038	0.0753
Subject 2	65.30 ± 1.11	28.30 ± 0.76	29.73 ± 0.47	<0.0001	0.0560
Subject 3	51.15 ± 1.16	10.19 ± 0.56	16.63 ± 0.52	<0.0001	<0.0001
Subject 4	57.85 ± 1.97	35.84 ± 1.15	37.12 ± 1.03	<0.0001	0.2038

[a]Refers to the mean percentage of shared spacers on skin compared against the mean percentage of spacers shared between saliva and skin

Supplemental Table 5. Subject-specific spacer comparisons

	**Intra-subject**[a]	**Inter-subject**[a]	**P-value**[b]
SGI			
Subject 1	99.31 ± 0.13	56.79 ± 29.22	**<0.0001**
Subject 2	95.48 ± 0.70	57.23 ± 29.18	**<0.0001**
Subject 3	94.25 ± 1.38	57.54 ± 29.13	**<0.0001**
Subject 4	86.05 ± 3.14	56.76 ± 29.23	0.2210
SGII			
Subject 1	93.56 ± 1.04	35.25 ± 32.60	**<0.0001**
Subject 2	98.95 ± 1.11	34.88 ± 32.42	**<0.0001**
Subject 3	99.28 ± 0.14	34.82 ± 32.58	**<0.0001**
Subject 4	57.66 ± 6.90	34.87 ± 32.34	0.2753

[a]Estimates based on the mean of 10,000 iterations. 1,000 random spacers were sampled per iteration. Estimates include the abundance of individual spacers that are shared between the saliva and skin.

[b]Empirical p-value based on the fraction of times the estimated percent shared spacers for intra-subject comparisons exceeds inter-subject comparisons

Supplemental Table 6. Estimated shared 16S rRNA OTUs in saliva and skin

	Saliva		Skin		Saliva vs skin
**Subject**	**Percent Shared[a]**	**p-value[b]**	**Percent Shared[a]**	**p-value[b]**	**Percent Shared[a]**
Subject 1	83.09 ± 6.55	**0.0112**	86.68 ± 9.76	**0.0132**	43.61 ± 21.72
Subject 2	79.34 ± 6.59	**0.0059**	61.75 ± 26.28	0.2407	35.73 ± 22.67
Subject 3	81.31 ± 7.79	**0.0512**	85.71 ± 9.32	**0.0088**	48.89 ± 21.17
Subject 4	85.78 ± 5.58	**0.0008**	85.61 ± 7.75	**0.0183**	39.16 ± 20.35

[a]Based on the mean of 10,000 iterations. 1,000 random OTUs were sampled per iteration
[b]Empirical p-value based on the fraction of times the estimated percent shared OTUs for comparisons within skin or saliva exceeds that between skin and saliva
