# Supplementary material for: Conservation of streptococcal CRISPRs on human skin and saliva
Source: BMC Microbiol. 2014 Jun 6;14:146. doi: 10.1186/1471-2180-14-146 (PMC4063239; doi:10.1186/1471-2180-14-146)
Supplement: Additional file 2: Figure S1 — Rarefaction analysis of CRISPR spacer groups in the saliva and on the skin of all subjects. Figure S2. Heatmaps of SGII CRISPR spacer groups in all subjects. Figure S3. SGII CRISPR spacer group heat matrices from all subjects. Figure S4. Conservation of CRISPR spacer content by time of day sampled. Figure S5. Conservation of CRISPR spacer content by time of day sampled. Figure S6. Percentage of SGI (Panel A) and SGII (Panel B) CRISPR spacers with homologues in the NCBI NR database. Figure S7. Percentage of SGI (Panel A) and SGII (Panel B) CRISPR spacers matching virome reads from the subjects in this study. Figure S8. Bar graphs representing the percentage of CRISPR spacers (±standard deviation) with matches in human skin, oral, and gut-derived metagenomes. Figure S9. Relative rates of newly identified CRISPR spacers in skin and saliva of all subjects. Figure S10. Principal coordinates analysis of bacterial OTUs based on 16S rRNA sequences for the skin and saliva of all subjects. Figure S11. Percentage of taxonomic assignments from the Genus Streptococcus in all subjects for saliva and skin. [file 1471-2180-14-146-S2.pdf]

Supplemental Figure 1

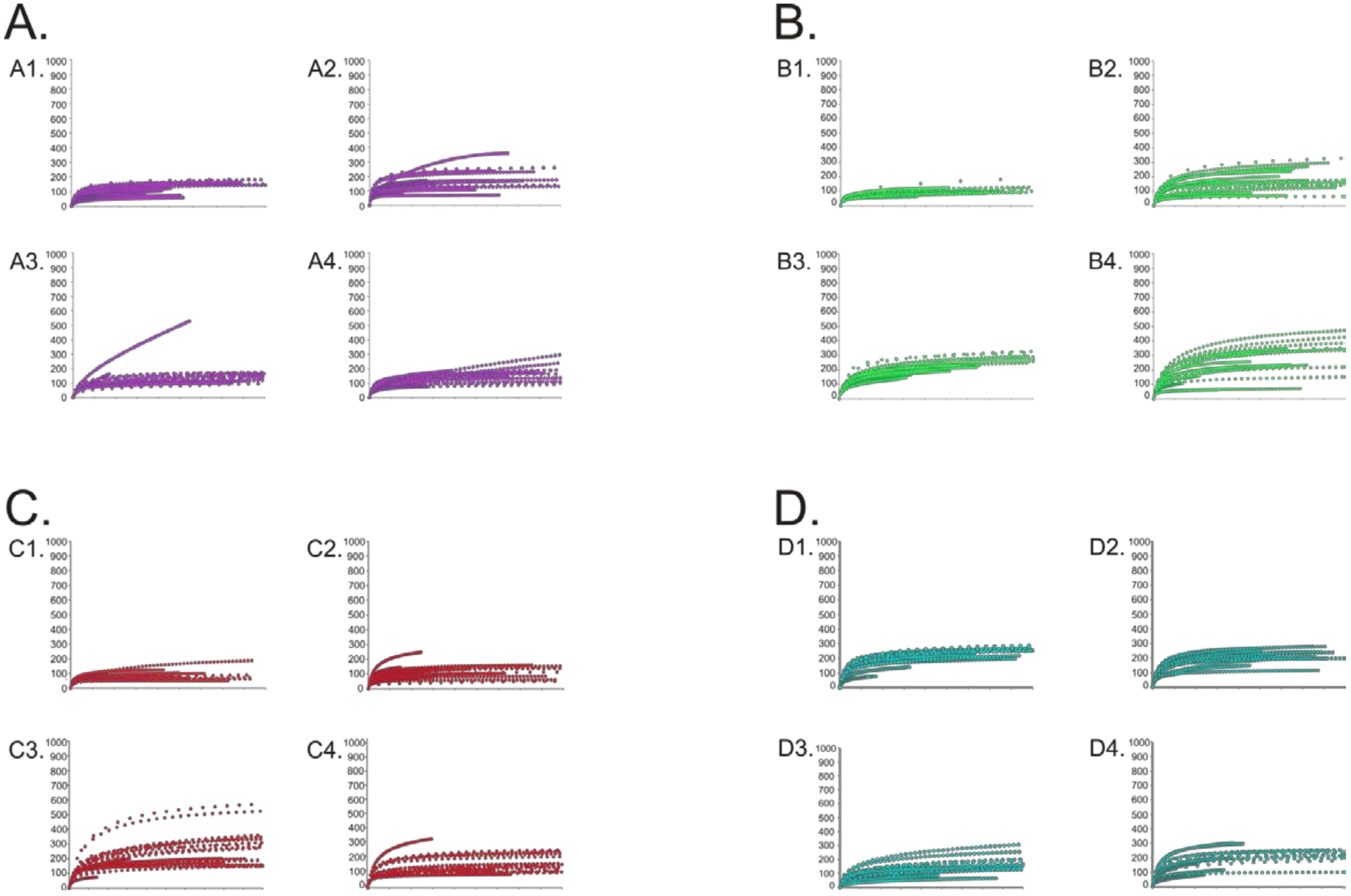

Supplemental Figure 1: Rarefaction analysis of CRISPR spacer groups in the saliva and on the skin of all subjects. Rarefaction curves were created using 10,000 random iterations based on spacer group richness. The y-axis represents the number of unique spacer groups and the x-axis represents the number of spacers sampled. Panel A – Subject1, Panel B – Subject #2, Panel C – Subject #3, and Panel D – Subject #4. Subpanels 1 and 2 represent SGI spacers and Subpanels 3 and 4 represent SGII spacers. Subpanels 1 and 3 also represent salivary CRISPRs, whereas Subpanels 2 and 4 also represent skin CRISPRs.

Supplemental Figure 2

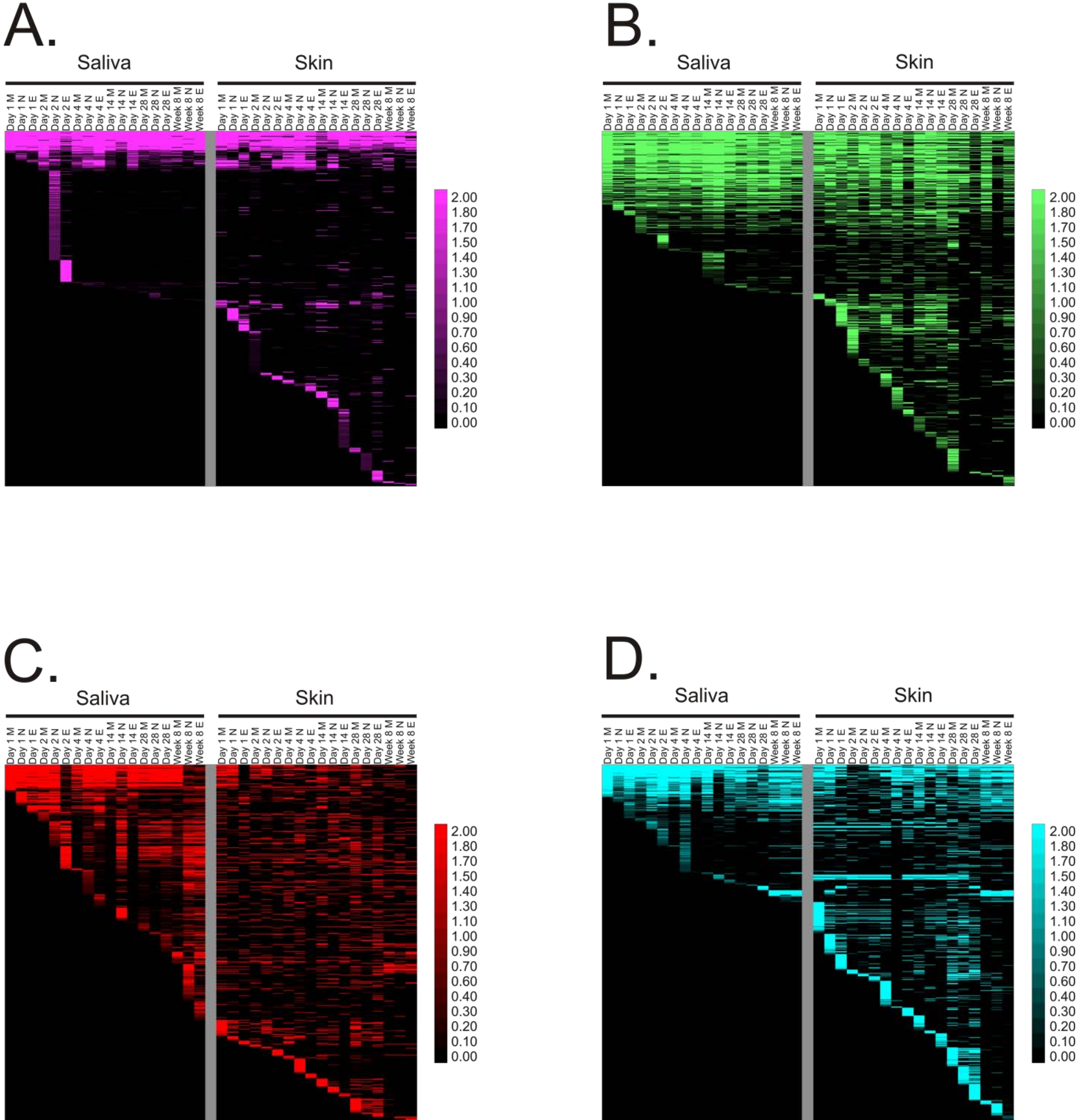

Supplemental Figure 2: Heatmaps of SGII CRISPR spacer groups in all subjects. Each row represents a unique spacer group and the columns represent each individual time point. Each day is listed, where M represents morning, N represents noon, and E represents evening. Saliva-derived SGII CRISPR spacer groups are demonstrated on the left, and skin-derived CRISPR spacer groups are on the right of each panel. The intensity scale bar is located to the right, and represents the percentage of total spacers found at each time point in each subject. Panel A – Subject #1, Panel B – Subject #2, Panel C – Subject #3, and Panel D – Subject #4.

Supplemental Figure 3

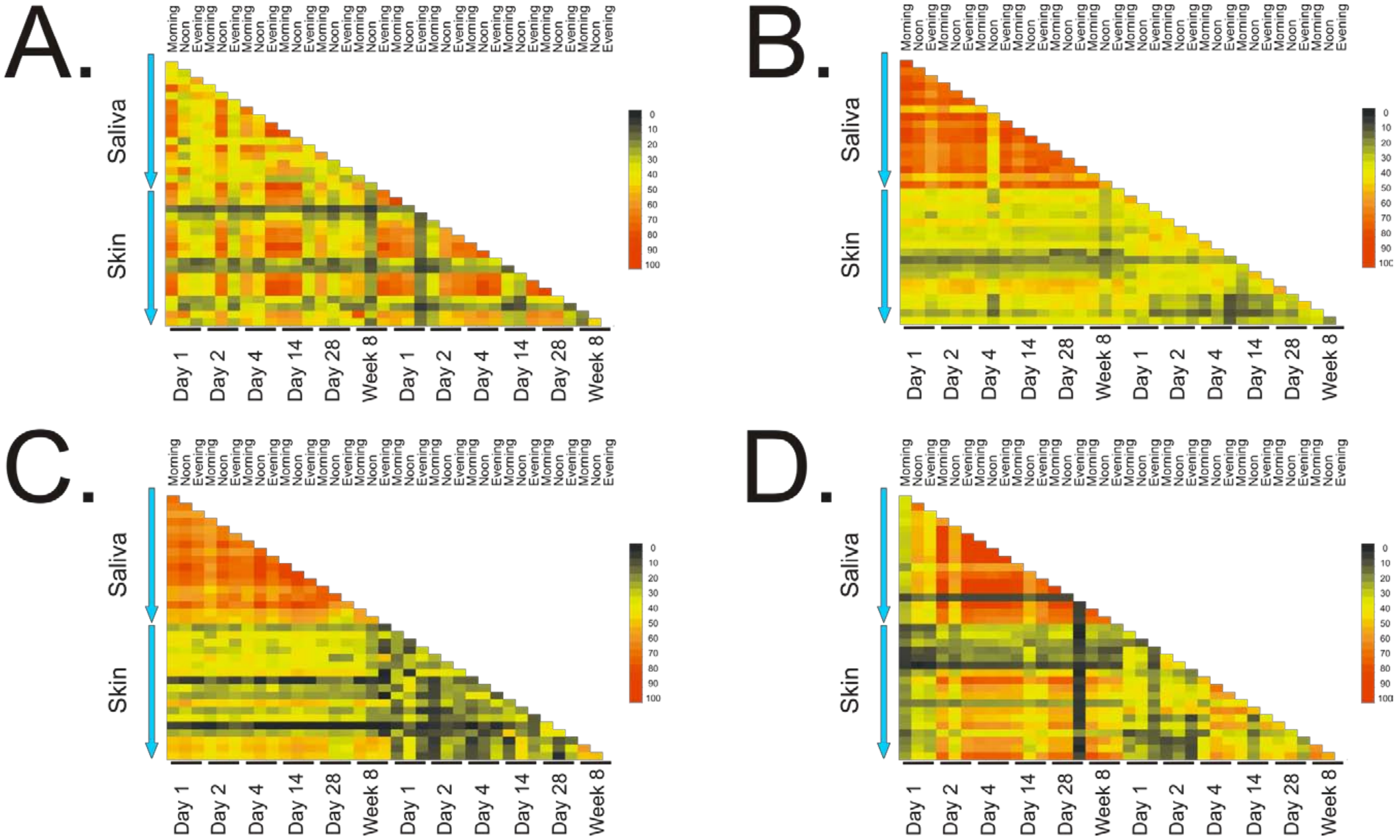

Supplemental Figure 3: SGII CRISPR spacer group heat matrices from all subjects. Each matrix demonstrates the percentage of shared SGII CRISPR spacer groups between all time points within each subject. The top triangular portion of each matrix represents comparisons between saliva-derived CRISPR spacers, the bottom rectangular portion of each matrix represents comparisons between saliva-derived and skin-derived CRISPR spacers, and the bottom triangular portion of each matrix represents comparisons between skin-derived CRISPR spacers. The intensity scale bar is located to the right of each matrix. Panel A – Subject #1, Panel B – Subject #2, Panel C – Subject #3, and Panel D – Subject #4.

Supplemental Figure 4

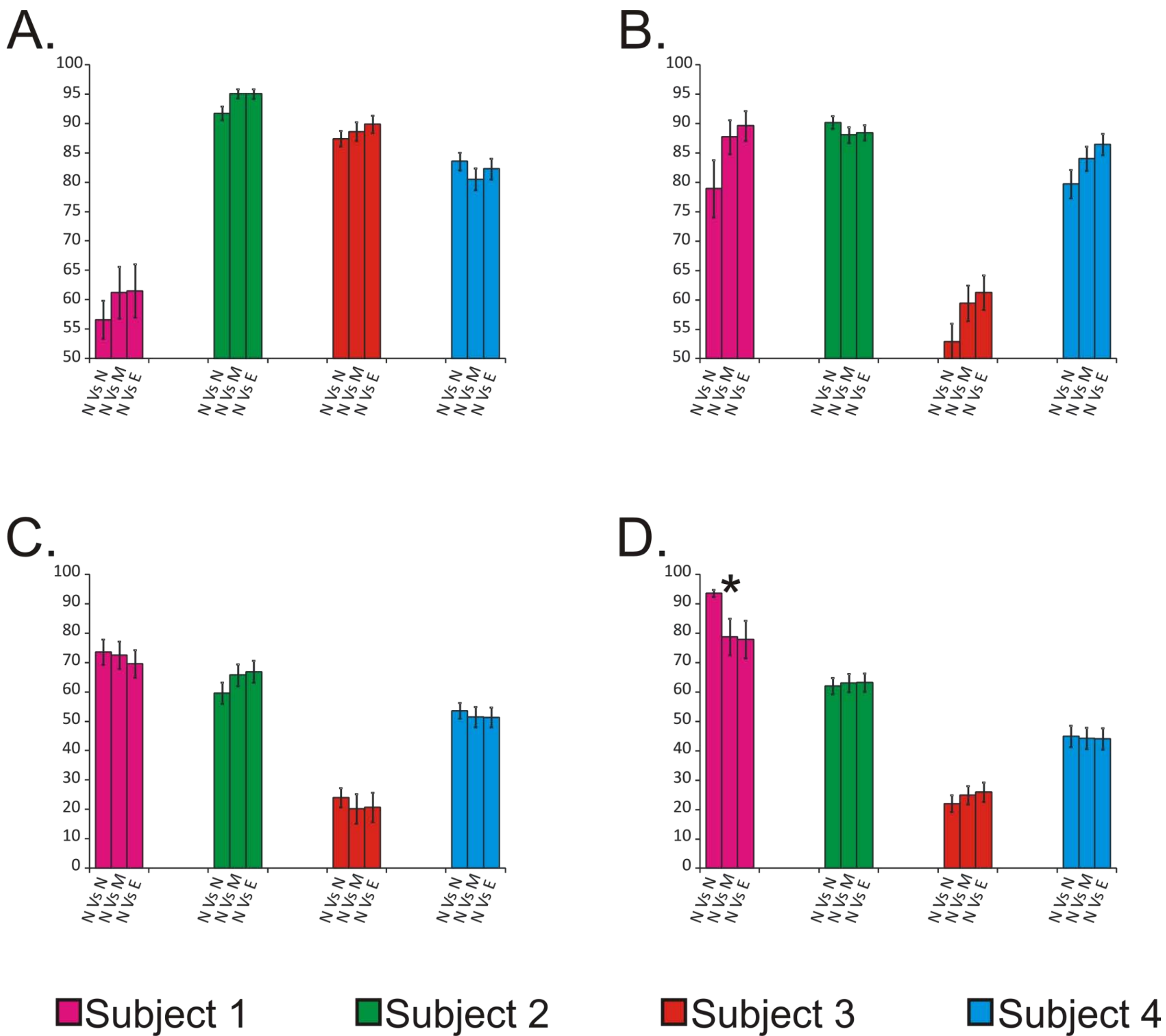

Supplemental Figure 4: Conservation of CRISPR spacer content by time of day sampled. Each panel demonstrates the relative conservation of spacers (± standard deviation) within the noon time points for each subject (N vs. N), comparisons of the noon time points with morning time points (N vs. M), and comparisons of the noon time points with the evening time points (N vs E) for subject #1 (magenta), subject #2 [22], subject #3 (red), and subject #4 (cyan). Panels A and B represent salivary SGII and SGI CRISPR spacers, respectively. Panels C and D represent skin-derived SGII and SGI CRISPR spacers, respectively. The '*' represents subjects in which the relative conservation of spacers for the noon time points is significantly ($p \leq 0.05$) greater than for comparisons of noon and morning/evening time points.

Supplemental Figure 5

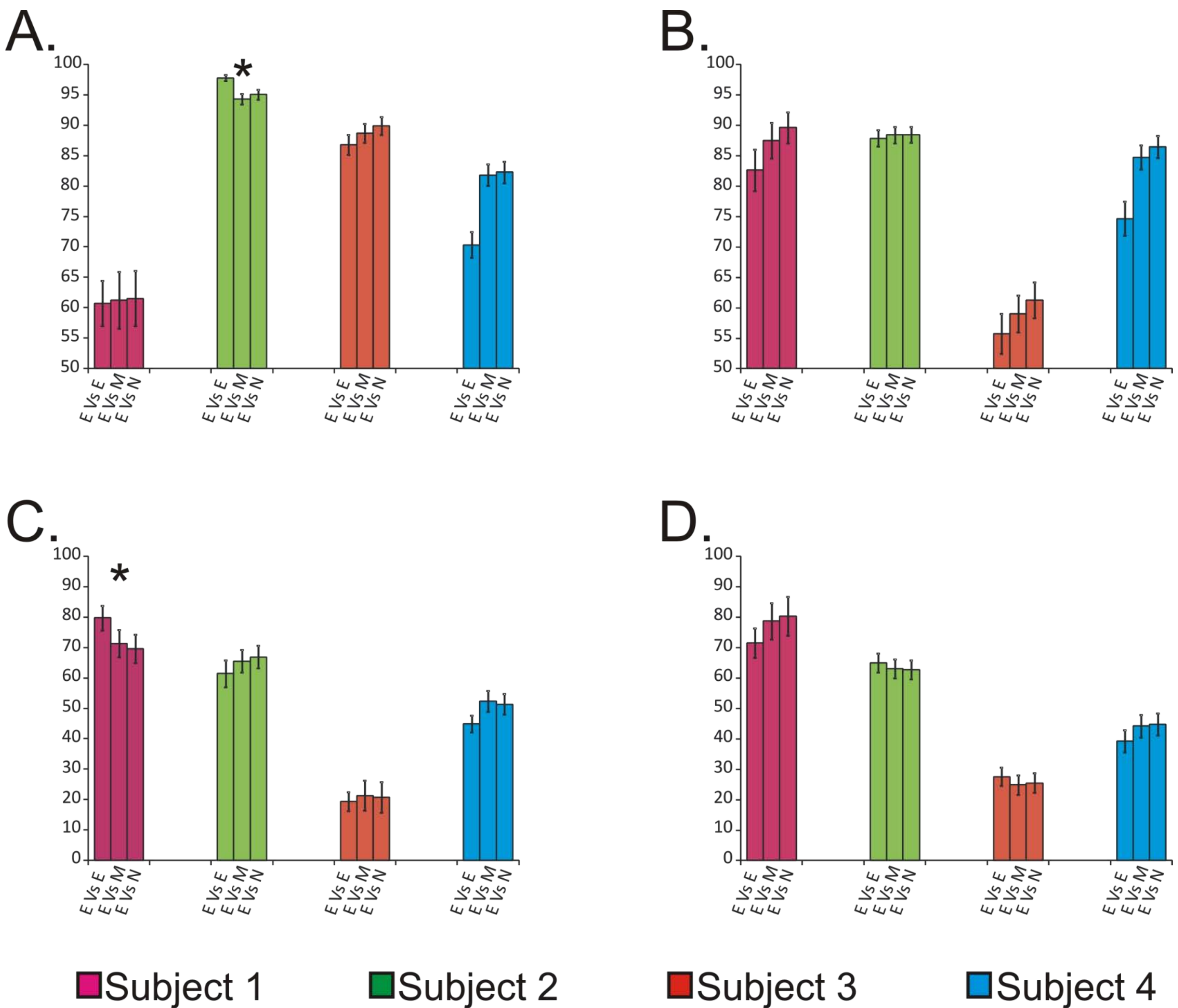

Supplemental Figure 5: Conservation of CRISPR spacer content by time of day sampled. Each panel demonstrates the relative conservation of spacers (± standard deviation) within the evening time points for each subject (E vs. E), comparisons of the evening time points with morning time points (E vs. M), and comparisons of the evening time points with the noon time points (E vs N) for subject #1 (magenta), subject #2 [22], subject #3 (red), and subject #4 (cyan). Panels A and B represent salivary SGII and SGI CRISPR spacers, respectively. Panels C and D represent skin-derived SGII and SGI CRISPR spacers, respectively. The '*' represents subjects in which the relative conservation of spacers for the evening time points is significantly ($p \leq 0.05$) greater than for comparisons of evening and morning/noon time points.

Supplemental Figure 6

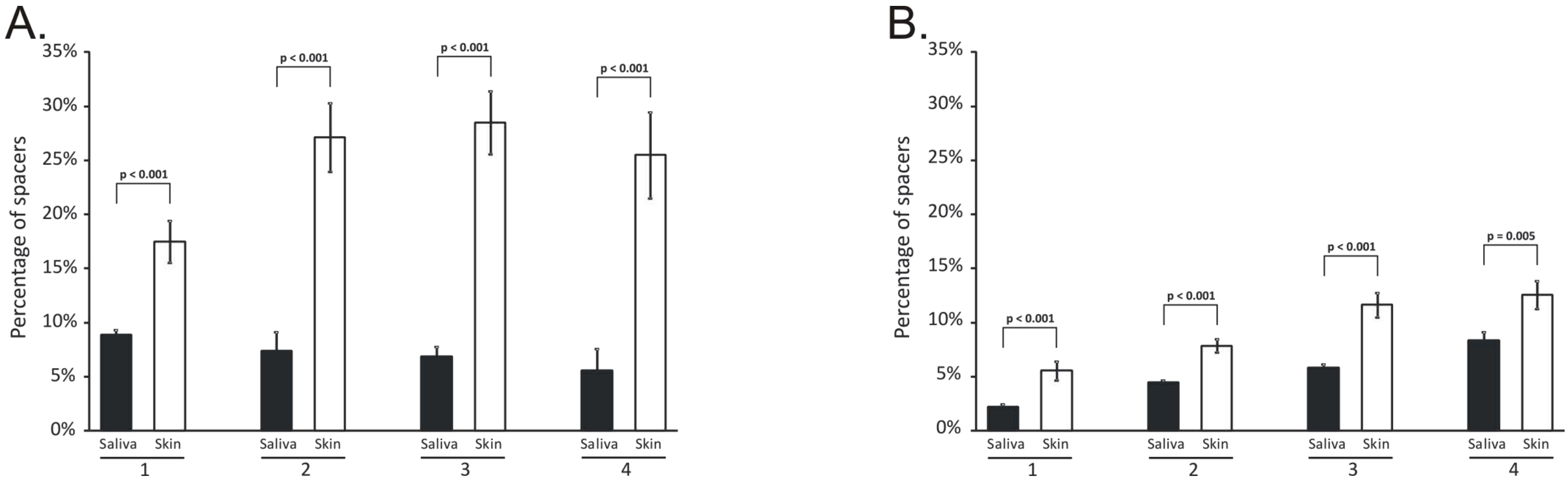

Supplemental Figure 6: Percentage of SGI (Panel A) and SGII (Panel B) CRISPR spacers with homologues in the NCBI NR database. The Y-axis shows the mean percentage of the CRISPR spacers from all time points combined that had viral homologues in the NR database. The X-axis represents the saliva- and skin-derived spacers for each subject. Standard error bars are represented above each bar, and the p-value is demonstrated above each error bar. Subjects 1 through 4 are shown consecutively from left to right on the X-axis.

Supplemental Figure 7

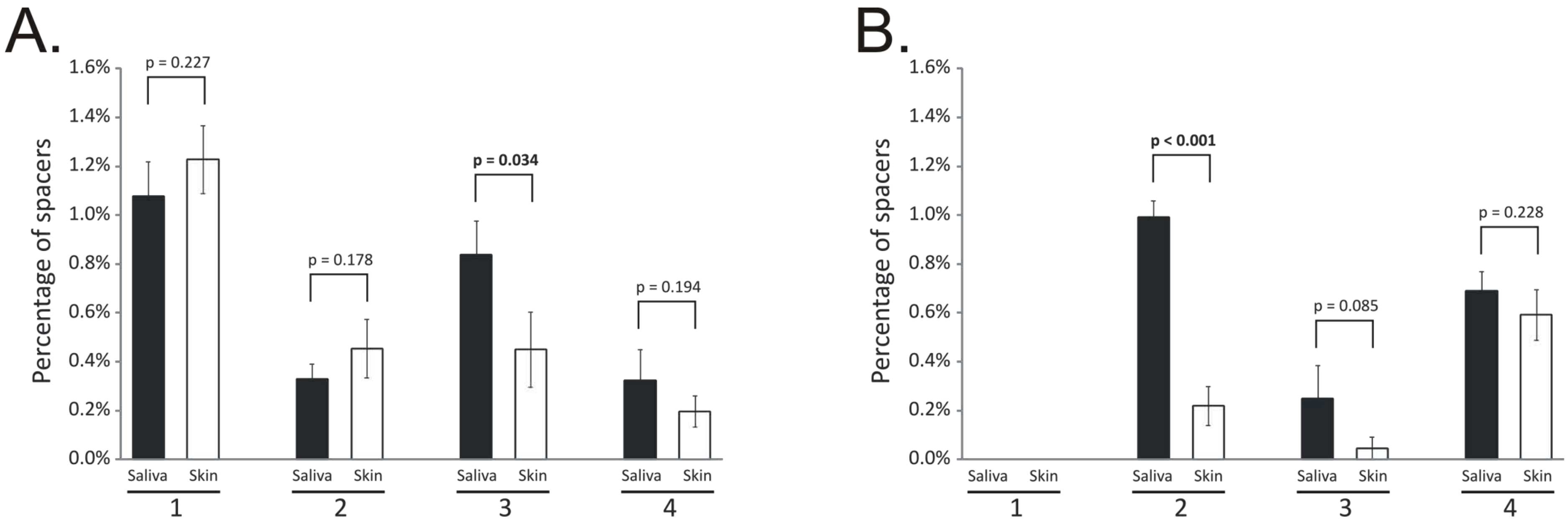

Supplemental Figure 7: Percentage of SGI (Panel A) and SGII (Panel B) CRISPR spacers matching virome reads from the subjects in this study. The Y-axis shows the mean percentage of the CRISPR spacers from all time points combined that matched virome reads from the subjects in this study. The X-axis represents the saliva- and skin-derived spacers for each subject. Standard error bars are represented above each bar, and the p-value is demonstrated above each error bar. Subjects 1 through 4 are shown consecutively from left to right on the X-axis.

Supplemental Figure 8

A.

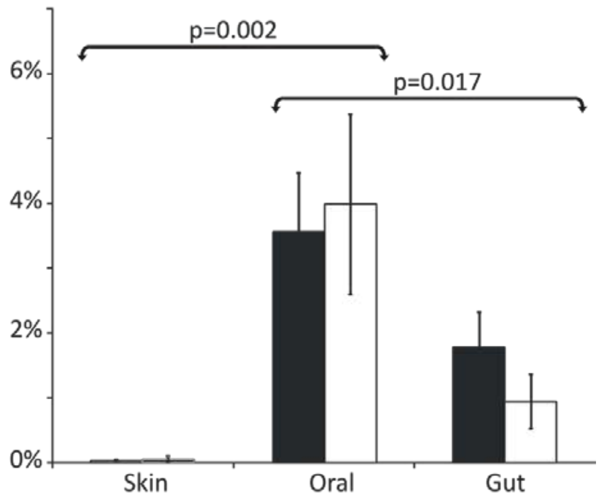

B.

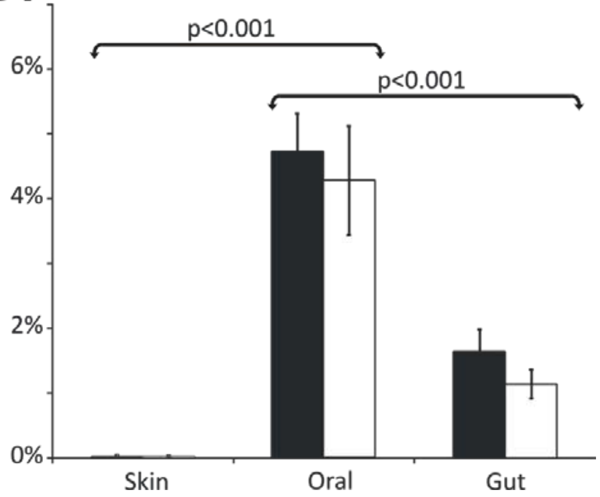

Supplemental Figure 8: Bar graphs representing the percentage of CRISPR spacers (± standard deviation) with matches in human skin, oral, and gut-derived metagenomes. Panel A – SGII spacers, and Panel B – SGI spacers. P-values from comparisons of skin vs oral metagenomes and gut vs oral metagenomes are demonstrated at the top of each panel.

Supplemental Figure 9

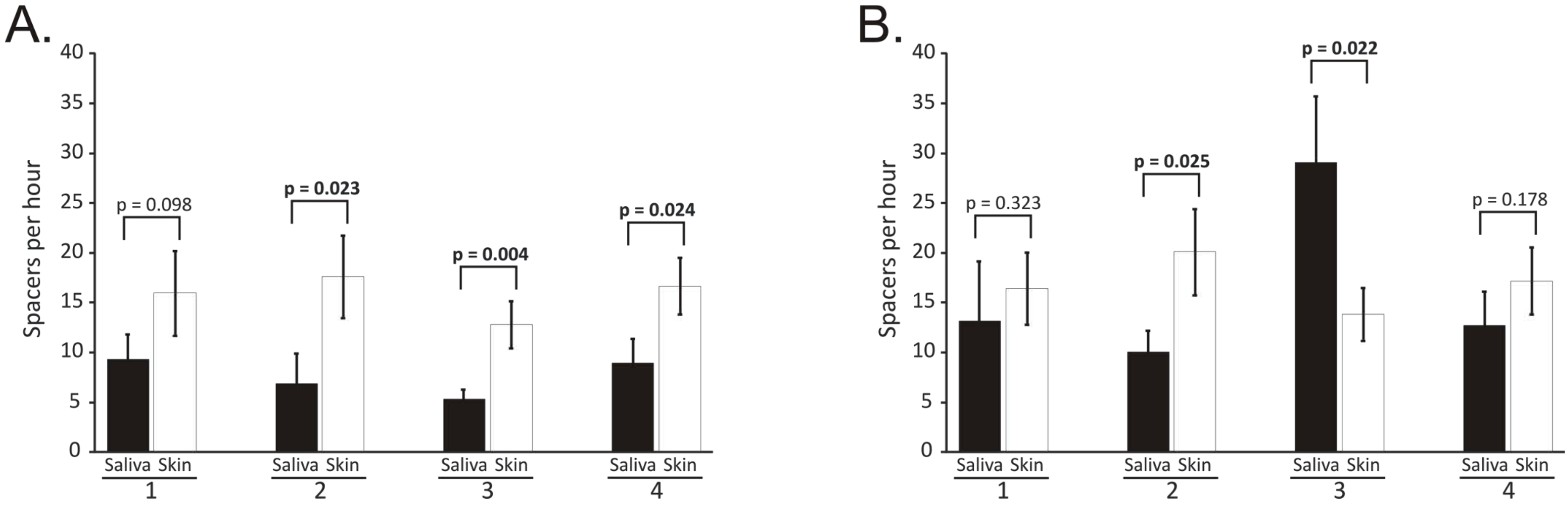

Supplemental Figure 9: Relative rates of newly identified CRISPR spacers in skin and saliva of all subjects. The Y-axis represents the estimated rate of newly identified spacers per hour. Standard error bars are represented above each bar, and the p-value is demonstrated above each error bar. Panel A represents SGII spacers and Panel B represents SGI spacers. Subjects 1 through 4 are shown consecutively from left to right on the X-axis.

Supplemental Figure 10

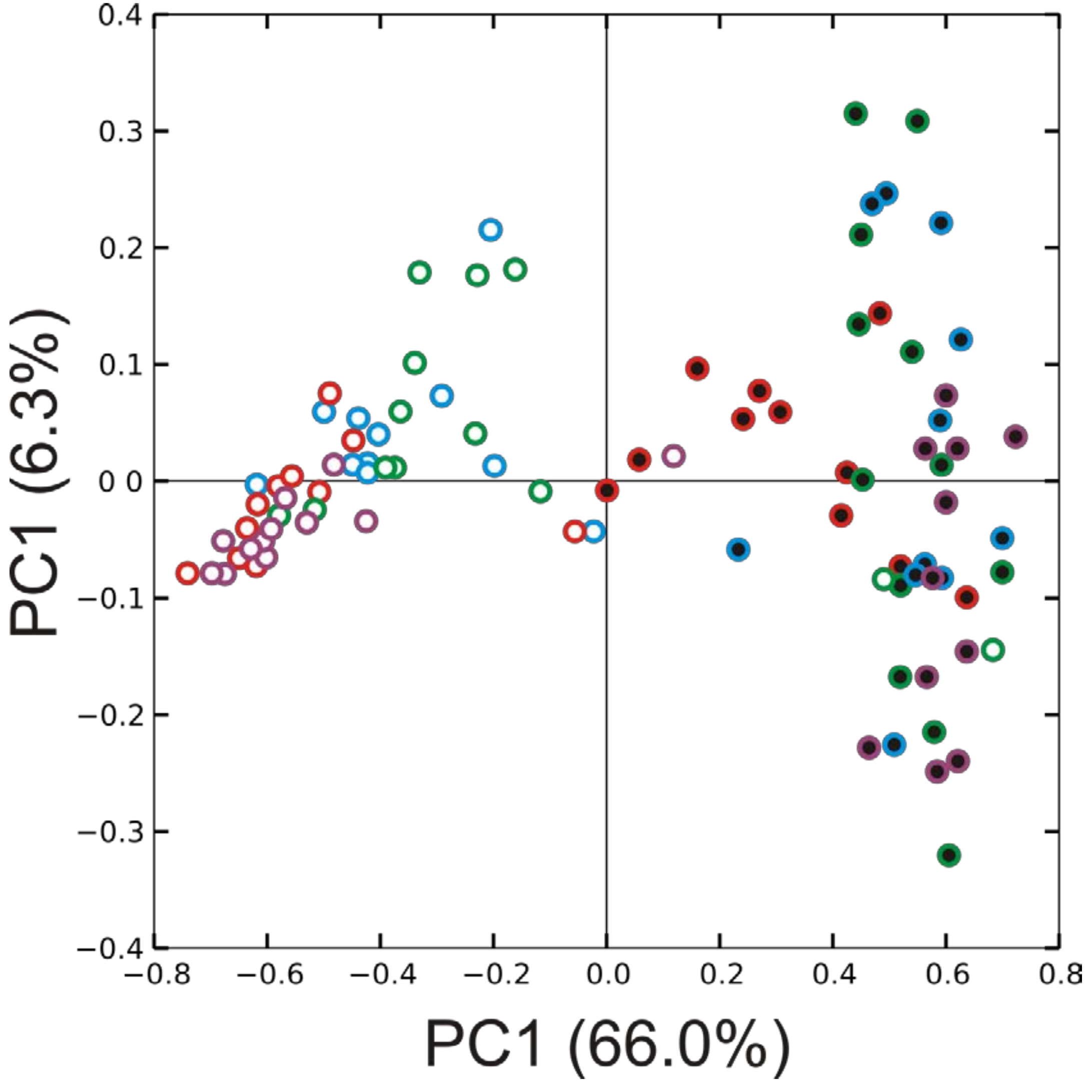

Supplemental Figure 10: Principal coordinates analysis of bacterial OTUs based on 16S rRNA sequences for the skin and saliva of all subjects. Beta diversity was determined using weighted Unifrac distances. The white inner circles represent skin-derived sequences and the black inner circles represent saliva-derived sequences. Subject #1 is represented by purple outer circles, Subject #2 is represented by green outer circles, Subject #3 is represented by red outer circles, and Subject #4 is represented by cyan outer circles.

Supplemental Figure 11

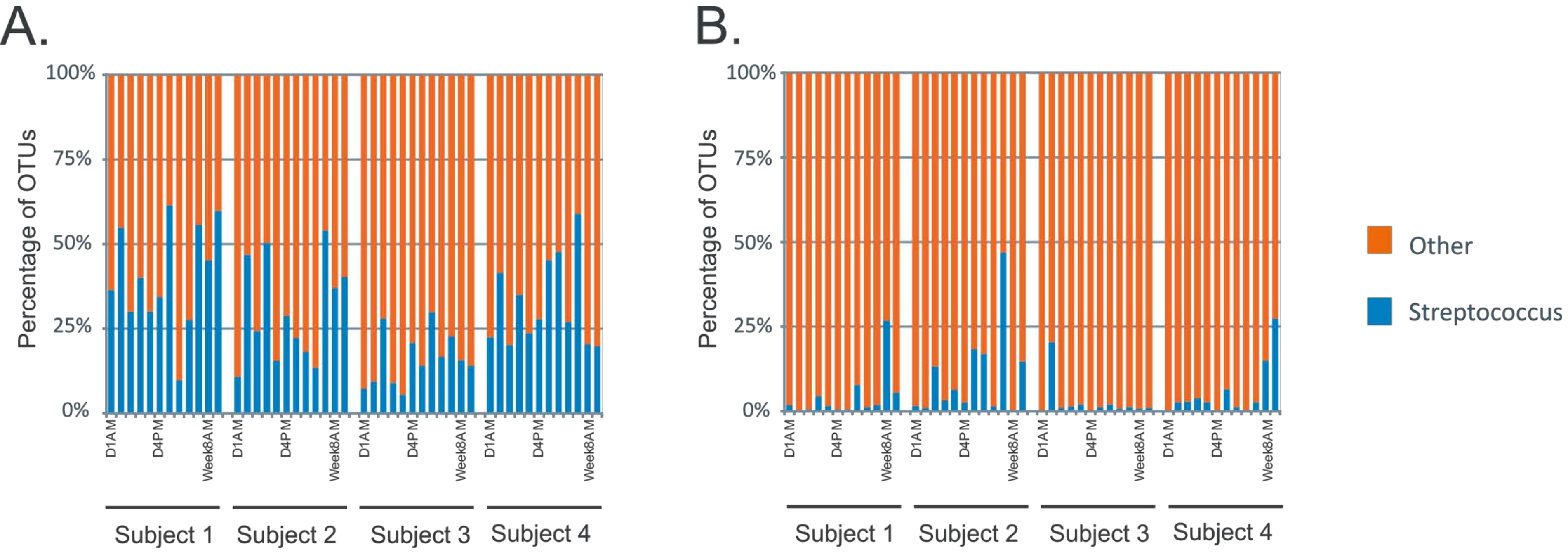

Supplemental Figure 11: Percentage of taxonomic assignments from the Genus *Streptococcus* in all subjects for saliva and skin. The percentage of 16S rRNA reads assigned to each group is demonstrated on the y-axis. Panels A and B represent the percentage of total reads that were assigned to the Genus *Streptococcus* from saliva (Panel A) and skin (Panel B).
